# Unveiling promising drug targets for autism spectrum disorder: insights from genetics, transcriptomics, and proteomics

**DOI:** 10.1093/bib/bbae353

**Published:** 2024-07-22

**Authors:** Rui Jiang, Wentao Huang, Xinqi Qiu, Jianyi Chen, Ruibang Luo, Ruijie Zeng, Shuangshuang Tong, Yanlin Lyu, Panpan Sun, Qizhou Lian, Felix W Leung, Yufeng Liu, Weihong Sha, Hao Chen

**Affiliations:** Department of Gastroenterology, Guangdong Provincial People's Hospital (Guangdong Academy of Medical Sciences), Southern Medical University, No. 106, Zhongshan 2nd Road, Guangzhou 510080, China; The Second School of Clinical Medicine, Southern Medical University, No. 1023 Shatainan Road, Guangzhou 510515, China; School of Medicine, South China University of Technology, No. 230, West Waihuan Road, Higher Education Mega Centre, Panyu District, Guangzhou 510006, China; Department of Gastroenterology, Guangdong Provincial People's Hospital (Guangdong Academy of Medical Sciences), Southern Medical University, No. 106, Zhongshan 2nd Road, Guangzhou 510080, China; The Second School of Clinical Medicine, Southern Medical University, No. 1023 Shatainan Road, Guangzhou 510515, China; Cancer Prevention Center, State Key Laboratory of Oncology in South China, Collaborative Innovation Center for Cancer Medicine, Sun Yat-sen University Cancer Center, No. 651 Dongfeng East Road, Guangzhou 510060, China; Department of Gastroenterology, Guangdong Provincial People's Hospital (Guangdong Academy of Medical Sciences), Southern Medical University, No. 106, Zhongshan 2nd Road, Guangzhou 510080, China; School of Medicine, South China University of Technology, No. 230, West Waihuan Road, Higher Education Mega Centre, Panyu District, Guangzhou 510006, China; Department of Computer Science, The University of Hong Kong, Pokfulam Road, Hong Kong 999077, China; Department of Gastroenterology, Guangdong Provincial People's Hospital (Guangdong Academy of Medical Sciences), Southern Medical University, No. 106, Zhongshan 2nd Road, Guangzhou 510080, China; Department of Gastroenterology, Guangdong Provincial People's Hospital (Guangdong Academy of Medical Sciences), Southern Medical University, No. 106, Zhongshan 2nd Road, Guangzhou 510080, China; Shantou University Medical College, Shantou University, No. 22 Xinling Road, Shantou 515041, Guangdong, China; Department of Gastroenterology, Guangdong Provincial People's Hospital (Guangdong Academy of Medical Sciences), Southern Medical University, No. 106, Zhongshan 2nd Road, Guangzhou 510080, China; Shantou University Medical College, Shantou University, No. 22 Xinling Road, Shantou 515041, Guangdong, China; Faculty of Synthetic Biology, Shenzhen Institute of Advanced Technology, Chinese Academy of Sciences, No. 1068 Xueyuan Avenue, Shenzhen University Town, Shenzhen 518055, China; Faculty of Synthetic Biology, Shenzhen Institute of Advanced Technology, Chinese Academy of Sciences, No. 1068 Xueyuan Avenue, Shenzhen University Town, Shenzhen 518055, China; Cord Blood Bank, Guangzhou Institute of Eugenics and Perinatology, Guangzhou Women and Children’s Medical Center, Guangzhou Medical University, No. 9 Jinsui Road, Guangzhou 510623, China; State Key Laboratory of Pharmaceutical Biotechnology, The University of Hong Kong, Pokfulam Road, Hong Kong 999077, China; Sepulveda Ambulatory Care Center, VA Greater Los Angeles Healthcare System, 16111 Plummer Street, Los Angeles 91343, California, United States; University of California Los Angeles David Geffen School of Medicine, 10833 Le Conte Avenue, Los Angeles 90095, California, United States; Center for Medical Research on Innovation and Translation, Guangzhou First People's Hospital, the Second Affiliated Hospital of South China University of Technology, No 1 Panfu Road, Guangzhou 510000, China; Department of Gastroenterology, Guangdong Provincial People's Hospital (Guangdong Academy of Medical Sciences), Southern Medical University, No. 106, Zhongshan 2nd Road, Guangzhou 510080, China; The Second School of Clinical Medicine, Southern Medical University, No. 1023 Shatainan Road, Guangzhou 510515, China; School of Medicine, South China University of Technology, No. 230, West Waihuan Road, Higher Education Mega Centre, Panyu District, Guangzhou 510006, China; Shantou University Medical College, Shantou University, No. 22 Xinling Road, Shantou 515041, Guangdong, China; Department of Gastroenterology, Guangdong Provincial People's Hospital (Guangdong Academy of Medical Sciences), Southern Medical University, No. 106, Zhongshan 2nd Road, Guangzhou 510080, China; The Second School of Clinical Medicine, Southern Medical University, No. 1023 Shatainan Road, Guangzhou 510515, China; School of Medicine, South China University of Technology, No. 230, West Waihuan Road, Higher Education Mega Centre, Panyu District, Guangzhou 510006, China; Shantou University Medical College, Shantou University, No. 22 Xinling Road, Shantou 515041, Guangdong, China

**Keywords:** Mendelian randomization, autism spectrum disorder, drug target prediction

## Abstract

Autism spectrum disorder (ASD) is a complex neurodevelopmental disorder for which current treatments are limited and drug development costs are prohibitive. Identifying drug targets for ASD is crucial for the development of targeted therapies. Summary-level data of expression quantitative trait loci obtained from GTEx, protein quantitative trait loci data from the ROSMAP project, and two ASD genome-wide association studies datasets were utilized for discovery and replication. We conducted a combined analysis using Mendelian randomization (MR), transcriptome-wide association studies, Bayesian colocalization, and summary-data-based MR to identify potential therapeutic targets associated with ASD and examine whether there are shared causal variants among them. Furthermore, pathway and drug enrichment analyses were performed to further explore the underlying mechanisms and summarize the current status of pharmacological targets for developing drugs to treat ASD. The protein–protein interaction (PPI) network and mouse knockout models were performed to estimate the effect of therapeutic targets. A total of 17 genes revealed causal associations with ASD and were identified as potential targets for ASD patients. Cathepsin B (CTSB) [odd ratio (OR) = 2.66 95, confidence interval (CI): 1.28–5.52, *P* = 8.84 × 10^−3^], gamma-aminobutyric acid type B receptor subunit 1 (GABBR1) (OR = 1.99, 95CI: 1.06–3.75, *P* = 3.24 × 10^−2^), and formin like 1 (FMNL1) (OR = 0.15, 95CI: 0.04–0.58, *P* = 5.59 × 10^−3^) were replicated in the proteome-wide MR analyses. In Drugbank, two potential therapeutic drugs, Acamprosate (GABBR1 inhibitor) and Bryostatin 1 (CASP8 inhibitor), were inferred as potential influencers of autism. Knockout mouse models suggested the involvement of the *CASP8*, *GABBR1*, and *PLEKHM1* genes in neurological processes. Our findings suggest 17 candidate therapeutic targets for ASD and provide novel drug targets for therapy development and critical drug repurposing opportunities.

## Introduction

Autism spectrum disorder (ASD) is a neurodevelopmental disorder characterized by impairments in social interaction, communication, and restricted repetitive patterns of behavior that primarily affect children. ASD sufferers have higher rates of death compared to the general population, particularly those with additional mental and physical health conditions [1]. The global prevalence of ASD is estimated at 1%, especially in children [2]. The economic burden of ASD is substantial. Many existing medications primarily target specific aspects of ASD, such as managing repetitive behaviors or addressing communication difficulties, leaving a considerable gap in addressing the holistic spectrum of symptoms, and individual responses to these medications can vary significantly [3]. Therefore, there is a need for research on novel drug targets to address this condition.

Genetic factors play an important role in the pathogenesis of ASD, and the occurrence of ASD is likely associated with variations in multiple genes. Grove *et al*. [4] report a genome-wide association meta-analysis and identified candidate genes, including *NEGR1*, *PTBP2*, *CADPS*, *KCNN2*, *KMT2E*, and *MACROD2*. Although the genome-wide association studies (GWAS) have identified many single nucleotide polymorphisms (SNPs) linked to ASD, whether these SNP’s nearest genes can be directly used as drug targets is uncertain. With gene sequence development, we could detect more incorporating genetics in drug development, which could be one of the most efficient ways to improve the process, because drugs with genetic support are considerably more likely to succeed in clinical trials [5].

With the advancement of high-throughput genomics and proteomics technologies in plasma and cerebrospinal fluid, the strategy based on Mendelian randomization (MR) has facilitated the identification of potential therapeutic targets for numerous diseases and the application of drug repurposing [6]. Analogous methodologies have been employed to investigate diseases, such as Parkinson’s [7], COVID-19 [8], and multiple sclerosis [9]. MR leverages naturally occurring genetic variants as instrumental variables to infer drug efficacy by imitating random trials, combining the SNP-gene-expression (or associated with medication targeting the encoded protein) and the SNP-disease associations (extracted from GWAS data) to provide insights into potential therapeutic targets. Colocalization methods examine the overlap in genetic signals between two distinct traits, such as gene expression and disease susceptibility. By identifying shared genetic loci, colocalization helps pinpoint genes associated with both traits, shedding light on potential causal relationships [10]. Significant MR results could confirm the pathogenicity of drug targets, not just as biomarkers.

**Figure 1:**
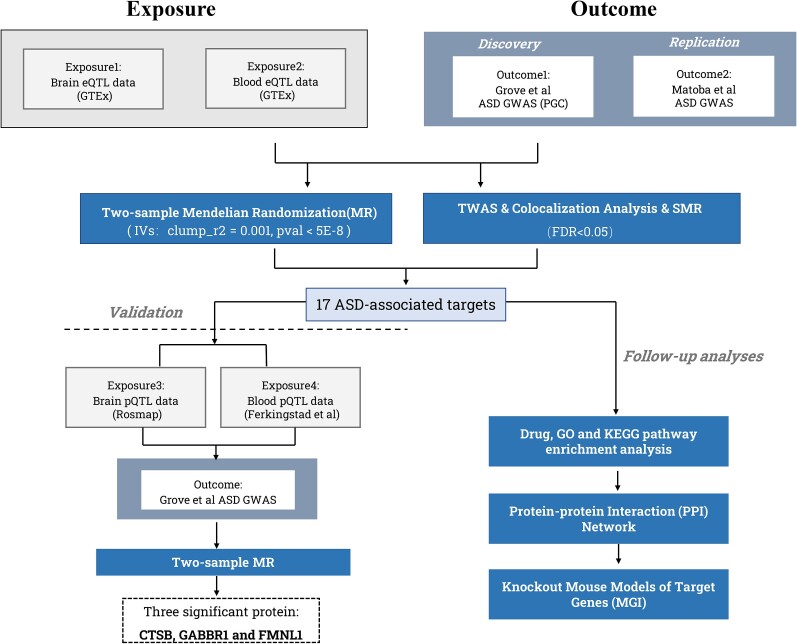
Overview of the study design. MR, Mendelian randomization; ASD, autism spectrum disorder; eQTL, expression quantitative trait loci; pQTL, protein quantitative trait loci.

In this study, we conducted a comprehensive analysis using MR and colocalization techniques to identify causal drug targets for ASD and filling the existing gaps in ASD treatments. Our analysis utilizes genetic variants associated with quantitative expression trait loci (eQTLs) or protein quantitative trait loci (pQTL) as exposures to estimate the causal relationships between risk factors and diseases. Furthermore, we explored the potential mechanisms of action associated with these drug targets through enrichment analysis (Fig. 1, Supplemental Table S1). By employing these methods, we aimed to gain insights into the genetic basis of ASD and identify promising therapeutic targets with a better understanding of their underlying mechanisms. This deeper comprehension can guide the development of novel medications that address the specific genetic and biological factors contributing to ASD, potentially leading to more targeted and effective interventions.

## Materials and methods

### Gene expression and proteomic data source

The eQTL data were sourced from Genotype-Tissue Expression (GTEx) project v8, which comprises 54 non-diseased tissue sites across nearly 1000 individuals. We applied a significant cis-eQTL (*P* < 5 × 10^−8^) filtering criterion, resulting in 5887 genes in 13 brain regions and 1956 genes in whole blood that matched ASD GWAS data and were thus included in MR analysis. The pQTL summary statistics were obtained from the religious orders study and memory and aging project (ROSMAP) (*n* = 376) [11]. A total of 912 253 SNP-protein expression pairs were included in the ROSMAP pQTL dataset, and we extracted significant SNPs (pQTL *P* < 5 × 10^−8^). Another summary-level statistics of genetic associations with levels of 4907 circulating proteins were extracted from a large-scale pQTL study in 35 559 Icelanders [12]. Proteomics profiling was performed by a multiplexed, modified aptamer-based binding assay (SOMAscan version 4). We performed linkage disequilibrium (LD) clumping (*r*^2^ < 0.001) to get an independent association.

### GWAS summary statistics of ASD

In the discovery stage, summary-level data for ASD were available in the authoritative Psychiatric Genomic Consortium (PGC), a genome-wide association meta-analysis of 18 381 individuals with ASD and 27 969 controls [4]. Other summary-level ASD GWAS data for validation was obtained from the GWAS catalog, containing 22 916 European ancestry cases and 32 504 European ancestry controls [13].

### Mendelian randomization analysis

MR analyses were completed using the R package ‘TwoSampleMR’. In this study, the eQTL and pQTL data for brain and blood tissues were used as genetic instrumental variables, and the autism GWAS summary statistics were used as the outcome. The exposure and outcome data were loaded and harmonized using in-built functions. SNPs were then clumped (*r*^2^ < 0.001, windows = 10 000 kb) using European samples from the 1000 Genomes Project reference panel. Wald ratios were calculated for those genes with one SNP. Where >2 SNPs were available per exposure, inverse-variance-weighted MR was conducted. In the discovery study and replication studies, we deemed FDR < 0.05 to be significant, and we identified the common genes from both as candidate targets. The three assumptions of MR are as follows. First, the SNPs are strongly associated with the exposure (*P* < 5 × 10^−8^). Second, the SNPs are independent of confounding factors. Third, the SNPs are only related to the outcome through the exposure.

### Transcriptome-wide association studies (TWAS)

Transcriptome-wide association studies (TWAS) integrate genotype data with gene expression profiles to identify genes whose expression levels are associated with a particular phenotype. In this study, we employed TWAS FUSION software [14] to investigate the genetic basis of autism. Based on gene expression levels from GETx V8 13 brain tissues and summary statistics from genome-wide association analysis of PGC ASD. The results were adjusted for potential confounders and multiple testing using false discovery rate (FDR) control.

### Bayesian co-localization analysis

We carried out a colocalization analysis for ASD outcomes using the R package ‘coloc’. Based on the Bayesian method, there are five exclusive hypotheses: (i) no association with either trait; (ii) association with trait 1 only; (iii) association with trait 2 only; (iv) both traits are associated, but distinct causal variants were for two traits; and (v) both traits are associated, and the same shares a causal variant for both traits [15]. In the colocalization analysis, we merged ASD GWAS data with eQTL expression data for 17 candidate targets obtained from MR. We therefore restricted our analysis to genes reaching posterior probability of hypothesis 3 (PPH3) + PPH4 ≥ 0.8 [7], and PPH4 > 0.8 is considered to indicate a strong co-localization association.

### Summary-data-based Mendelian randomization (SMR) analysis

We conducted a summary-data-based Mendelian randomization (SMR) analysis to investigate the causal effect of 17 candidate gene expressions on ASD. Thirteen brain tissues of eQTL summary statistics were obtained from the GTEx V8 project, while ASD GWAS summary statistics were sourced from PGC. SNPs significantly associated with gene expression (*P* < 5 × 10^−8^) were selected as instrumental variables. SMR associations were deemed significant if they exceeded an FDR threshold of <0.05 and passed the heterogeneity in dependent instruments (HEIDI) outlier test (*P* > 0.05, with a minimum of >10 SNPs).

### Functional enrichment analysis

Gene ontology (GO) analysis and Kyoto Encyclopedia of Genes and Genomes (KEGG) enrichment analysis were conducted to gain insights into the biological processes, cellular components, molecular functional roles, and pathways associated with the identified 17 autism-target genes. We searched the DrugBank database to obtain drug development information for identified targets and inquired about their primary functions. Additionally, we also searched for any research linking these existing drugs to ASD.

### Protein–protein interaction (PPI) network

Based on the Open Targets database, we extracted the susceptibility-associated proteins of ASD identified in the previous analyses, and taking into account those with drug scores >0.5. Subsequently, we examined the interactions between the 17 potential drug targets we identified and previously associated proteins related to ASD susceptibility. All PPI analyses were conducted using the Search Tool for 5 the Retrieval of Interacting Genes (STRING) database version 11.5 (https://string-db.org/). The results of PPI analysis were updated into Cytoscape software (ver.3.7.1) to visualize these interactions and construct a PPI network.

### Abnormal phenotypes in knockout mouse models of target genes

We utilized the international database resource for laboratory mice, Mouse Genome Informatics (MGI, http://www.informatics.jax.org/), to identify phenotypic markers and gene information associated with all 17 candidate genes related to ASD in knockout or mutant mice. Our focus primarily rested on abnormal phenotypes within the nervous system, aiming to explore the correlations with ASD.

## Results

### MR reveals 17 genes casually associated with ASD using brain and blood eQTL

**Figure 2:**
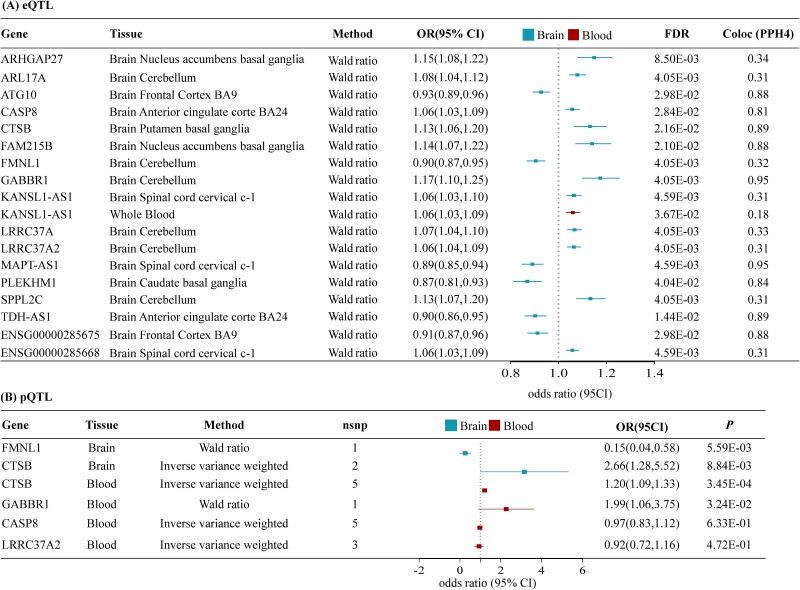
(A) Candidate ASD-risk genes identified by MR and colocalization in brain and blood eQTL. (B) Replicate candidate ASD-risk genes through brain and blood pQTL and ASD GWAS in MR.CI, confidence interval; ASD, autism spectrum disorder; OR: odds ratio; FDR, false discovery rate; PPH4, posterior probability of hypothesis 4.

Using the instrumental variables (IVs) derived from GTEx eQTL data in 13 brain tissues, we performed two-sample MR to identify the candidate therapeutic targets for ASD. We found significant MR results for 21 potential drug targets KANSL1 antisense RNA 1 (*KANSL1-AS1*), leucine rich repeat containing 37 member A2 (*LRRC37A2*), ENSG00000285668, ADP ribosylation factor like GTPase 17A (*ARL17A*), caspase 8 (*CASP8*), TDH antisense RNA 1 (*TDH-AS1*), leucine rich repeat containing 37A (*LRRC37A*), family with sequence similarity 215 member B (*FAM215B*), pleckstrin homology and RUN domain containing M1 (*PLEKHM1*), autophagy related 10 (*ATG10*), MAPT antisense RNA 1 (*MAPT-AS1*), ribosomal protein S23 (*RPS23*), Rho GTPase activating protein 27 (*ARHGAP27*), cathepsin B (*CTSB*), formin like 1 (*FMNL1*), gamma-aminobutyric acid type B receptor subunit 1 (*GABBR1*), signal peptide peptidase like 2C (*SPPL2C*), ENSG00000236234, ENSG00000255310, ENSG00000265547, and ENSG00000285675 in at least one tissue in the IPSYCH ASD GWAS (Supplementary Table S2, available online at http://bib.oxfordjournals.org/). A total of 17 genes were validated in the replication ASD GWAS dataset from Matoba *et al*. (Fig. 2A and Supplementary Table S3, available online at http://bib.oxfordjournals.org/). All significant MR estimates were identified with a Benjamini–Hochberg FDR of <0.05. Notably, in the discovery and replication analysis, significant genetic association signals (*TDH-AS1*, *ATG10*, and *FMNL1*) might be casual protective association with the risk of ASD. The MR estimates for *ARHGAP27*, *LRRC37A2*, *ARL17A*, *CASP8*, *CTSB*, *FAM215B*, *GABBR1*, *SPPL2C*, *KANSL1-AS1*, *LRRC37A*, and ENSG00000285668 were consistently positive and associated with increased ASD risk in all significant tissues (Supplementary Table S3, available online at http://bib.oxfordjournals.org/).

### ASD-association genes identified through transcriptome-wide association study analysis

Of the 17 candidate targets identified in MR, TWAS analysis revealed 13 genes, including ARHGAP27, ARL17A, ATG10, CASP8, CTSB, KANSL1-AS1, LRRC37A, LRRC37A2, MAPT-AS1, PLEKHM1, SPPL2C, TDH-AS1, and FAM215B, and 79 features significantly associated with the ASD of interest exhibited the strongest associations with FDR < 0.05 (Supplemental Table S4).

### Candidate therapeutic targets for ASD identified by Colocalization and SMR

Colocalization analysis between ASD risk genes and eQTL in the brain was performed as a sensitivity analysis for MR to provide more strong evidence for associations and shared genetic effects between target genes and ASD. Our results identified that all 17 genes with sufficient power (PPH3 + PPH4 ≥ 0.8, Supplementary Table S5, available online at http://bib.oxfordjournals.org/) and revealed strong evidence of colocalization (PPH4 > 0.8) for nine targets, including *MAPT-AS1*, *GABBR1*, *CASP8*, *TDH*-*AS1*, *CTSB*, *ATG10*, *FAM215B*, *PLEKHM1*, and ENSG00000285675 (Fig. 2A). *KANSL1*-*AS1* was the only overlapping gene between the brain and whole blood. SMR analysis identified 12 genome-wide significant associations (pSMR < 0.05), of which nine survived the HEIDI-outlier test (Supplementary Table S6, available online at http://bib.oxfordjournals.org/). In summary, our SMR results align closely with the colocalization findings, indicating a consistent pattern of association.

### Replicated target genes in brain and blood pQTL data

Using conditional independent brain and blood pQTL data, we applied the two-sample MR to further validate our proposed drug targets at the protein level. For all 17 genes obtained above, only two proteins (*CTSB* and *FMNL1*) in the brain and four proteins (*CTSB*, *LRRC37A2*, *CASP8*, and *GABBR1*) in the blood were analyzed. Our results determined that a high level of *CTSB* increased the risk of ASD (brain: odds ratio [OR] = 2.66; 95% CI, 1.28–5.52; *P* = 8.84 × 10^−3^; blood: OR = 1.20; 95% CI, 1.09–1.33; *P* = 3.45 × 10^−4^; Fig. 2B). We also evaluate casual evidence for two proteins, *GABBR1* (blood: OR = 1.99; 95% CI, 1.06–3.75; *P* = 3.24 × 10^−2^) and *FMNL1* (brain: OR = 0.15; 95% CI, 0.04–0.58; *P* = 5.59 × 10^−3^), overlapped with the ASD-risk genes.

### Functional enrichment analysis of the ASD-risk genes

We conducted KEGG and GO enrichment to evaluate evidence for significant (FDR < 0.05) pathways enriched by these ASD-risk target genes. For KEGG, four pathways were significantly enriched, indicating that these target genes affected a series of important pathological processes, pathways of autophagy, apoptosis, Salmonella infection, and the NOD-like receptor signaling pathway (Supplementary Fig. S1). For GO enrichment analysis, we identified 22 significantly enriched terms for the cellular component (Supplementary Fig. S2), the cell periphery enriched by the largest number of target genes. Twenty-two terms for molecular function were significantly enriched (Supplementary Fig. S3), with endopeptidase activity, peptidase activity, Atg12 transferase activity, and cysteine-type endopeptidase activity emerging as the most significant.

### Evaluation of druggability of candidate targets

To evaluate the drug development activities of candidate target genes, we searched for 17 targets through the DrugBank database. As summarized in Table 1, several existing drugs targeting *CTSB*, *CASP8*, and *GABBR1* were identified. We listed the identified drug targets, as well as related drugs for which these drug targets have been approved for clinical use in the treatment of ASD. Acamprosate and Bryostatin 1, as inhibitors of GABBR1 and CASP8, respectively, provide more reliable evidence for the management and treatment of ASD. *ATG10*, *ARHGAP27*, *LRRC37A*, *FMNL1*, *SPPL2C*, *ARL17A*, *KANSL1*-*AS1*, *PLEKHM1*, *FAM215B*, *LRRC37A2*, *TDH*-*AS1*, *MAPT*-*AS*, ENSG00000285668, and ENSG00000285675 have not been investigated as specific drug targets and warranted further exploration as novel targets.

**Table 1:** Druggability of targets causally associated with ASDs and their research associations. CTSB, Cathepsin B; GABBR1, gamma-aminobutyric acid type B receptor subunit 1; CASP8, Caspase 8

Gene	Protein	DrugBank			PMID
		General function	Drug name	Molecular action	
CTSB	Cathepsin B	Proteoglycan binding	2-Aminoethanimidic acid		
			3-Amino-4-Oxybenzyl-2-Butanone		
			3-Methylphenylalanine		
			2-Pyridinethiol		
			Diphenylacetic acid		
			CA-074 methyl ester		35411983
			Trastuzumab deruxtecan	Substrate	
GABBR1	Gamma-Aminobutyric Acid Type B Receptor Subunit 1	G-protein coupled gaba receptor activity	Baclofen	Agonist	25754761
			Progabide		
			SGS-742		
			Tezampanel		
			Arbaclofen	Agonist	27748740
			Arbaclofen Placarbil	Agonist	
			gamma-Aminobutyric acid		
			Taurine	Agonist	
			Acamprosate	Inhibitor	25300441
			L-Baclofen	Agonist	
			Clozapine	Positive modulator	30285998
CASP8	Caspase 8	Ubiquitin protein ligase binding	Bardoxolone		
			Bryostatin 1	Inhibitor	33093534
			AN-9	Regulator	
			Trichostatin A	Activator	25133713
			Oleandrin	Regulator	

### Protein–protein interaction (PPI) network

**Figure 3:**
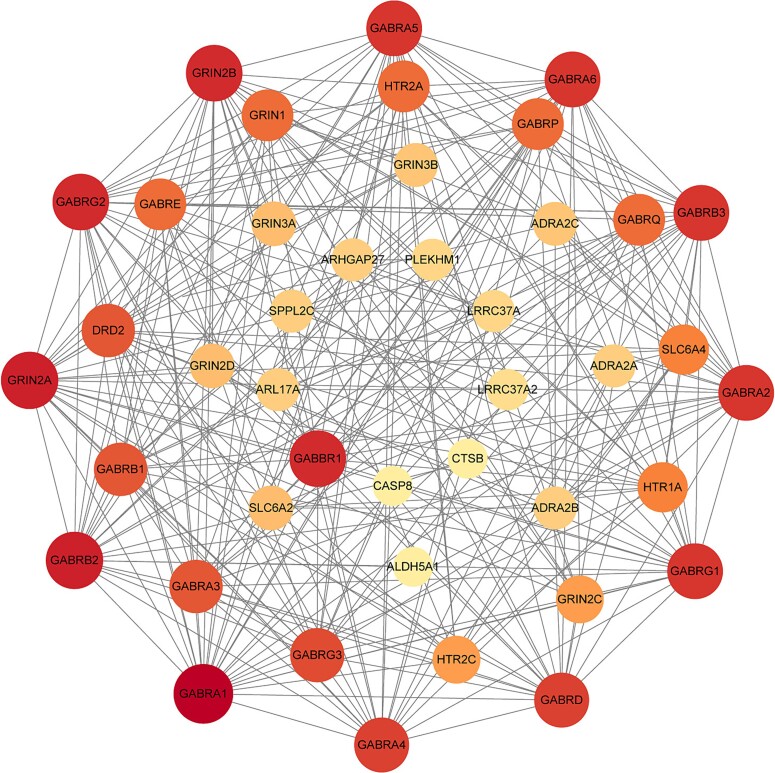
The protein–protein interaction network analysis based on candidate targets we identified (innermost circle) and susceptibility-associated proteins of ASD.

After excluding genes that could not be traced in the database and those that were independent and unrelated, a total of nine genes showed certain associations with the existing drug target genes for ASD (Fig. 3). The PPI network revealed GABBR1 as a prioritized protein interacting with various current ASD drug targets. Except for ATG10 and FMNL1, which showed no interactions with the previously identified targets in the PPI network, most of the other genes exhibited interactions with known targets. This suggests that our targets may function in similar biological processes, providing clues for the discovery of new drugs.

### Mouse knockout models of identified novel genes

We utilized the MGI resource to explore knockout mouse models, seeking evidence of phenotypic modifications associated with autism through target modifications. In the knockout or mutant models in mice, genes, such as CASP8, GABBR1, and PLEKHM1, exhibited various phenotypes related to neural system development and function, including wavy neural tube, abnormal neural tube morphology, kinked neural tube, abnormal optic disk morphology, and reduced long-term potentiation. Moreover, these genes were identified as direct human homologs, indicating their inherent roles in regulating neural system functions (Supplementary Table S7, available online at http://bib.oxfordjournals.org/).

## Discussion

ASD is a highly prevalent neurodevelopmental disorder, greatly influenced by genetics. While behavioral therapies and interventions can improve the quality of life for individuals with ASD, there is currently no cure for this complex condition. As a result, identifying new drug targets for the development of more effective pharmacological treatments is urgently required. MR and colocalization analysis led to the development of drugs for the management of coronary heart disease [6]. In this study, we utilized the MR, TWAS, colocalization analysis, and SMR to genetically identify 17 putative druggable genes associated with ASD. Three proteins *CTSB*, *GABBR1*, and *FMNL1* were replicated and showed a consistent effect on ASD risk between the pQTL and eQTL results. Enrichment analysis suggests that these genes might influence ASD mechanisms through lysosomes. The DrugBank database indicated three already-developed drug targets (*CTSB*, *CASP8*, and *GABBR1*). Acamprosate and Bryostatin 1, as inhibitors of GABBR1 and CASP8, respectively, might provide potential drug repurposing opportunities for ASD.

In order to identify potential target genes associated with ASD, we jointly utilized MR and Bayesian colocalization analyses to respectively infer the causality between quantitative trait loci (eQTL and pQTL) and ASD risk, as well as genetically estimate the shared genetic effect between them. Compared to identifying risk loci through a standalone GWAS analysis, these distinct but complementary methods could pinpoint the most likely functional variants and the genes they regulate, and they are increasingly being utilized in drug target selection. Compared to the study by Hickman *et al.* [16] and Dominguez-Alonso *et al.* [17] our research primarily focuses on translating these targets into ASD treatment, not only advancing potential drug targets for future development but also employing enrichment analysis and PPI studies to investigate the mechanisms and highly associated inhibitors or drugs, potentially providing insights for drug repurposing. Autism is considered a developmental neurodevelopmental disorder, and its onset may be related to changes in genes during development. Microglia in the developing brain strongly express the *CTSB*, and abnormalities in the *CTSB* may affect the powerful phagocytosis capacity in the early stages of brain development when pruning is most needed [18]. Individuals with GABBR1 missense variants exhibit neurodevelopmental motor and language delays.

In the validation analysis, utilizing TWAS facilitates a robust phenotype association analysis, confirming the validation of most genes and consistent expression patterns across brain regions. This indicates a higher likelihood of gene variations within specific brain areas. SMR, similar to colocalization, demonstrates the coherence between both sets of results, emphasizing the role of genes in ASD susceptibility across varied analytical frameworks. These findings underscore the multifaceted nature of ASD genetics, highlighting the necessity of integrating diverse methodologies for comprehensive understanding and validation.

In the primary MR analysis results, we applied the Wald ratio method when there is only one SNP and inverse variance weighted method when there are two or more SNPs. The findings demonstrate that 17 genes showed causal associations with the risk of ASD in both sets of GWAS data, and the colocalization results confirm whether the causal association signal shares the same variable [19]. Of these, eight genes (*CASP8*, *ARHGAP27*, *CTSB*, *LRRC37A*, *LRRC37A2*, *KANSL1*-*AS1*, *GABBR1*, and *MAPT*-*AS1*) have been previously reported in genetic studies [20–22] to be associated with the risk of ASD and were confirmed by our MR results. In addition, the remaining nine genes (*ATG10*, *FMNL1*, *SPPL2C*, *ARL17A*, *PLEKHM1*, *FAM215B*, *TDH*-*AS1*, ENSG00000285675, and ENSG00000285668) were suggested as completely novel drug targets. We observed that the directionality of targets in the brain tissue is generally consistent, except for the gene PLEKHM1, which exhibits inconsistent directionality in brain cerebellum and caudate basal ganglia. We interpret this result as indicative of the spatial specificity of PLEKHM1 in brain tissue [23]. Therefore, it is important to take into account the tissue-specific characteristics of PLEKHM1 when considering therapeutic approaches targeting this specific target.

*KANSL1-AS1*, a member of the lncRNA class 1, also known as *KANSL1* Antisense RNA 11, emerges as a noteworthy RNA gene in our findings, exhibiting significance in both brain and blood tissues. *KANSL1*-*AS1*, *MATP*-*AS1*, *LRRC37A*, and *LRRC37A2*, are located at 17q21.31 on chromosome 17, a locus closely associated with ASDs [21] and various neurodegenerative diseases including frontotemporal dementia and progressive supranuclear palsy [24]. The expression of *KANSL1*-*AS1*, *LRRC37A*, and *LRRC37A2* in the fetal brain was significantly associated with ASD [22]. *LRRC37A* and *LRRC37A2* are two members of the *LRRC37* gene family, which is a group of protein-coding genes involved in neuron-specific function [25].

In our results, both eQTL and pQTL analyses identified elevated expression of *CTSB* as a risk factor for ASD. Colocalization analysis also provided strong evidence (PPH4 = 0.89), identifying that the association is driven by the same causal variants underlying ASD. *CTSB*, a potent lysosomal protease, plays a significant role in multiple neurodegenerative disorders and has been associated with enhancing memory function. The expression of *CTSB* is significantly elevated in various brain tissues and neutrophils [26]. Differential expression of *CTSB* can lead to differences in neurogenesis and neuronal cell death. *CTSB* affects the nervous system by regulating neuronal synaptic plasticity, and the inhibitors of cathepsin B have the potential to be the therapeutic agents for traumatic brain injury [27]. Pain *et al.* [21] revealed a significant association between the gene-level feature for *CTSB* and ASD, and *CTSB* was also identified as an ASD candidate gene using protein interaction networks [28]. Our findings further substantiate the role of CTSB protein as a drug target for ASD in both brain and blood tissues. *GABBR1* is an important receptor for gamma-aminobutyric acid (GABA), functional impairment of GABA receptors is associated with neurodevelopmental disorders, and GABA alterations are widely present in many regions and areas of the autistic brain [29]. *CASP8* is a potent lysosomal protease that influences processes, such as apoptosis, immunogenic necrotic cell death, and pyroptosis. *CASP8* is involved in the development of the dopaminergic system during embryogenesis, and it has been linked to mild ASD-like behaviors, as well as neurodegenerative pathology and/or traumatic brain injury [30]. *ARHGAP27* was identified as a candidate gene for ASD through TWAS and mRNA profiling [31].

For novel target genes, *FMNL1* is implicated in immune processes, impacting T-cell migration and autoimmune disorders [32]. Given the strong association between ASD and parental autoimmune disorders, exploring drugs targeting this specific gene could provide valuable insights from an immunological perspective. *SPPL2C*, *ARL17A*, *PLEKHM1*, and *FAM215B* are both located at the 17q21.31 locus contribute to the development and manifestation of ASD. The presence of an inversion polymorphism at this locus further underscores its relevance to ASD and other neurodevelopmental disorders. The important autophagy ATG10 protein is down-regulated in models of autism, and basal neuron autophagy is deficient, impairing normal spinal cord pruning and social behavior during development [33]. In addition, autophagy can also participate in the growth and development of neurons [34]. *TDH*-*AS1*, ENSG00000285675, and ENSG00000285668 have been poorly researched, illustrating their potential for exploration and further investigation.

We observed significant enrichment of lysosomes (GO:0005764 and GO:0008233), autophagy (KEGG:04140), and apoptosis (KEGG:04210) in target genes. Dysregulation of lysosomal function has been implicated in several neurodegenerative disorders, including ASD [35]. Recent studies have uncovered a potential link between lysosomal GO functions and the pathogenesis of ASD. Cathepsin D, a major lysosomal protein significantly expressed in the brains of ASD patients, blocks neurite outgrowth in cultured neurons by regulating lysosomal trafficking and remodeling [36]. Autophagy fuses with lysosomes *in vivo* and contributes to neural development and regulating synaptic plasticity [37]. Synaptic plasticity demonstrates potential as a therapeutic target for single-gene disorders associated with autism [38]. These findings suggest that aberrant lysosome activity may be contributing to the pathophysiology of ASD, potentially through modulation of autophagy or protein turnover processes. Peptidase participates in neuronal as well as protein degradation and metabolism; its influence on neurotransmitters and synaptic transmission may result in neurological dysfunction and autistic symptoms [39]. Glycosaminoglycans, crucial components of the neural extracellular matrix, including the pia mater basement membrane, are integral to neurodevelopment [40]. Their altered expression underpins the genesis of ASDs, potentially affecting brain structure and developmental memory processes. Perturbations of GABA have been observed in the cerebellum, cingulate cortex, and fusiform gyrus of autistic patients, influencing neurotransmission during brain development [41].

We obtained target-medicine information from DrugBank on three existing drug targets (*CTSB*, *CASP8*, and *GABBR1*) currently under investigation or already on the market. While these drugs may have been originally developed for purposes other than autism treatment, our results suggest that medications targeting these specific points, especially target inhibitors, hold promise for potential repurposing in autism therapeutics. Acamprosate, as an inhibitor of *GABBR1*, exhibited significant improvement in social relatedness and inattention/hyperactivity disorder in 67% of patients in a clinical trial in adolescents with ASD [42]. Bryostatin 1, identified as an inhibitor of *CASP8*, has been confirmed to have therapeutic potential for various neurological disorders. It demonstrates the capability to rescue disrupted phenotypes in adult fragile X mice, improve hippocampal synapses, spatial learning, and memory. Furthermore, it ameliorates autism-like phenotypes and other behaviors in mice, indicating a potential role in the treatment of fully developed autism in the brain [43].

In the mouse gene knockout model, the deletion of the GABBR1 gene is associated with various neurological dysfunctions. Specifically, the abnormal axon morphology distorts the structure and functional development of brain hemispheric connections, impacting the complete development of the brain in autism patients. This distortion leads to the incomplete development of higher-order cognition and sensory functions [44]. Insights from embryonic studies on autism suggest early-stage impairments, even during the neural tube phase, where CASP8 may play a role at this profound phenotypic level. The defective pre-pulse inhibition phenotype [45] observed in high-functioning autism patients is validated in PLEKHM1 knockout models.

Our study also has several limitations. First, all related data included in our analyses were of European ancestry. While this provides valuable insights into the genetic basis of the studied traits within this specific population, it is important to acknowledge that the findings may not be directly applicable or generalizable to other ethnic groups. We are eager to acquire data from additional populations, particularly eQTL and pQTL data, for further validation and generalization of our results. Obtaining data from diverse populations would be highly valuable, as it would allow us to assess the broader applicability of our findings. Second, although we identified a considerable number of target genes at the eQTL level, our ability to further validate these findings based on pQTL is limited to only a small subset of genes due to the scarcity of available protein indicators. In the MR analysis, we employed the strictest criteria for the analysis, and the final results were presented using the Wald method. Due to the limited number of SNPs, sensitivity analysis could not be performed. In response to this limitation, we utilized diverse and independent data from multiple tissues to ensure the reliability of the results. Furthermore, we conducted a genotype-based study, and future studies are warranted to further elucidate our results *in vitro* and *in vivo* experiments to clarify the potential biological mechanisms of ASD, as well as validate them in clinical trials.

## Conclusion

In summary, the application of the novel approach of drug-target MR and Bayesian colocalization, we identified 17 potential drug targets associated with the pathogenesis of ASD. *CTSB*, *FMNL1* proteins prioritize actionable novel drug targets for development, and the inhibitor of *GABBR1* provides potential drug repurposing opportunities. Our findings suggest a potential role in the etiology or regulation of disease progression, providing important targets for the development of novel medications. Understanding the biological mechanisms underlying the identified targets in autism and their significance in disease occurrence and progression is of paramount importance.

**Key Points**

- Our findings suggest 17 candidate therapeutic targets for ASD and provide persuasive genetic evidence and specific targets to prioritize for drug reuse and development.
- Analyzing at the levels of gene and protein expression, and employing a combination of Mendelian randomization and colocalization methods to identify potential drug targets.
- Strengthening target gene reliability through subsequent functional analysis, suggesting potential for repurposing existing drugs.

## Supplementary Material

Supplementary_Figure_S1_bbae353

Supplementary_Figure_S2_bbae353

Supplementary_Figure_S3_bbae353

Supplemental_Table_S1_bbae353

Supplemental_Table_S2_bbae353

Supplemental_Table_S3_bbae353

Supplemental_Table_S4_bbae353

Supplemental_Table_S5_bbae353

Supplemental_Table_S6_bbae353

Supplemental_Table_S7_bbae353

Supplemental_Materials-revised_bbae353

## Data Availability

eQTL dataset: GTEXv8: https://eqtlgen.org/ pQTL dataset: Brain: ROSMAP: https://doi.org/10.7303/syn23627957 [11]. Blood: SOMAscan version 4: https://www.decode.com/summarydata/ [12]. GWAS summary statistics for ASD are available by application from: PGC: https://www.med.unc.edu/pgc/results-and-downloads [4] and GWAS Catalog https://www.ebi.ac.uk/gwas/home with accession ID GCST010514 [13].
