# Supplementary material for: Unveiling promising drug targets for autism spectrum disorder: insights from genetics, transcriptomics, and proteomics
Source: Brief Bioinform. 2024 Jul 22;25(4):bbae353. doi: 10.1093/bib/bbae353 (PMC11262832; doi:10.1093/bib/bbae353)

**Supplementary Figure S1.** KEGG pathway enrichment analysis of ASD-risk targets with 4 enriched pathways. Purple represents ATG10, yellow represents CASP8, red represents CTSB and blue represents PLEKHM1.


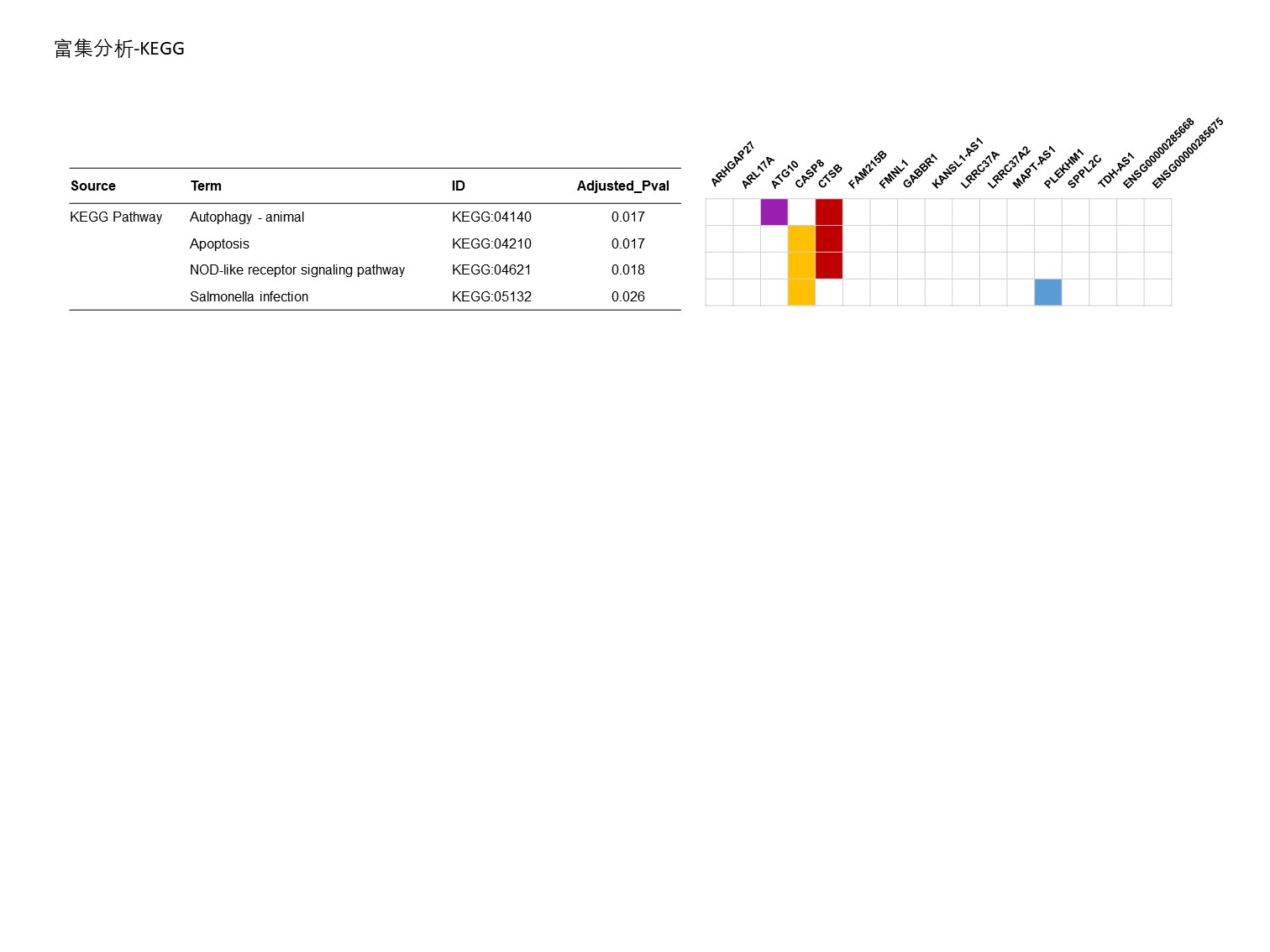

Supplement: Supplementary_Figure_S1_bbae353 [file supplementary_figure_s1_bbae353.docx]
