# Supplementary material for: Unveiling promising drug targets for autism spectrum disorder: insights from genetics, transcriptomics, and proteomics
Source: Brief Bioinform. 2024 Jul 22;25(4):bbae353. doi: 10.1093/bib/bbae353 (PMC11262832; doi:10.1093/bib/bbae353)

**Supplementary Figure S2.** GO Cellular Component enrichment analysis of ASD-risk targets with 22 enriched terms. Purple represents ATG10, yellow represents CASP8, red represents CTSB, cyan represents FAM215B, green represents GABBR1, blue represents PLEKHM1 and orange represents SPPL2C.


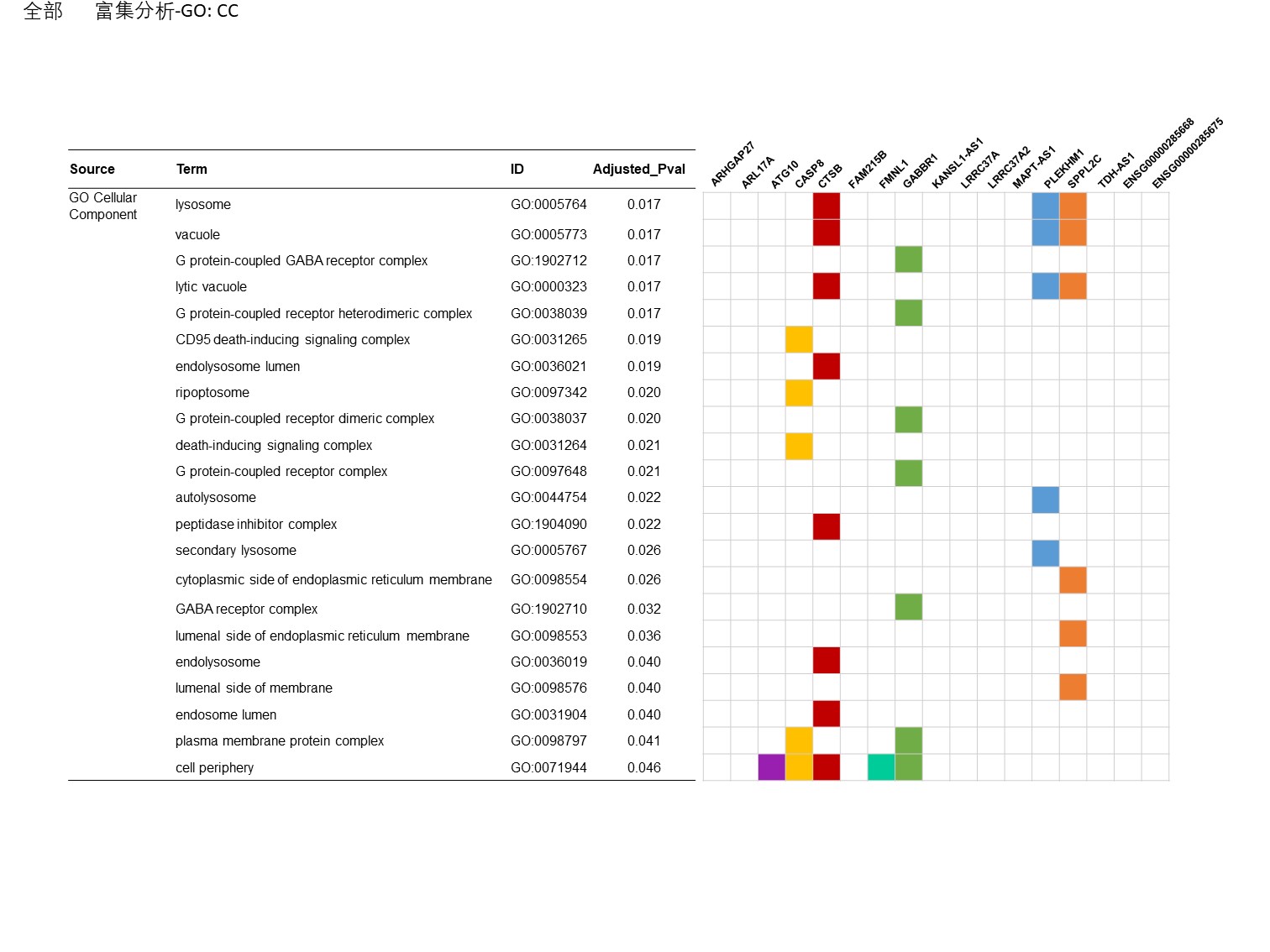

Supplement: Supplementary_Figure_S2_bbae353 [file supplementary_figure_s2_bbae353.docx]
