# Supplementary material for: Unveiling promising drug targets for autism spectrum disorder: insights from genetics, transcriptomics, and proteomics
Source: Brief Bioinform. 2024 Jul 22;25(4):bbae353. doi: 10.1093/bib/bbae353 (PMC11262832; doi:10.1093/bib/bbae353)
Supplement: Supplemental_Table_S1_bbae353 [file supplemental_table_s1_bbae353.docx]

**Supplemental Table S1**. Validation of 17 ASD targets in multiple methods.

| **Gene** | **MR-eQTL** | **MR-pQTL** | **Coloc** | **TWAS** | **SMR** |
| --- | --- | --- | --- | --- | --- |
| ARHGAP27 | **√** |  | **√** | **√** | **√** |
| ARL17A | **√** |  | **√** | **√** | **√** |
| ATG10 | **√** |  | **√** | **√** | **√** |
| CASP8 | **√** |  | **√** | **√** | **√** |
| **CTSB** | **√** | **√** | **√** | **√** | **√** |
| FAM215B | **√** |  | **√** | **√** | **√** |
| FMNL1 | **√** | **√** | **√** |  | **√** |
| GABBR1 | **√** | **√** | **√** |  | **√** |
| KANSL1-AS1 | **√** |  | **√** | **√** | **√** |
| LRRC37A | **√** |  | **√** | **√** |  |
| LRRC37A2 | **√** |  | **√** | **√** | **√** |
| MAPT-AS1 | **√** |  | **√** | **√** | **√** |
| PLEKHM1 | **√** |  | **√** | **√** | **√** |
| SPPL2C | **√** |  | **√** | **√** | **√** |
| TDH-AS1 | **√** |  | **√** | **√** |  |
| ENSG00000285675 | **√** |  | **√** |  |  |
| ENSG00000285668 | **√** |  | **√** |  |  |

MR, Mendelian Randomization; eQTL, expression quantitative trait loci; pQTL, protein quantitative trait loci; TWAS: transcriptome-wide association studies; SMR, Summary-data-based Mendelian Randomization.
