# Supplementary material for: Unveiling promising drug targets for autism spectrum disorder: insights from genetics, transcriptomics, and proteomics
Source: Brief Bioinform. 2024 Jul 22;25(4):bbae353. doi: 10.1093/bib/bbae353 (PMC11262832; doi:10.1093/bib/bbae353)
Supplement: Supplemental_Table_S2_bbae353 [file supplemental_table_s2_bbae353.docx]

**Supplemental Table S2. Estimated casual effects of eQTL on PGC ASD GWAS dataset using Mendelian Randomization.**

| **Gene** | **Tissue** | **method** | **OR (95CI)** | **Pvalue** | **FDR** |
| --- | --- | --- | --- | --- | --- |
| ARL17A | Brain Amygdala | Wald ratio | 1.11(1.06,1.17) | 5.59E-06 | 4.34E-03 |
| CASP8 | Brain Amygdala | Wald ratio | 1.08(1.03,1.12) | 2.24E-04 | 3.64E-02 |
| ENSG00000285668 | Brain Amygdala | Wald ratio | 1.07(1.04,1.10) | 2.30E-05 | 7.44E-03 |
| KANSL1-AS1 | Brain Amygdala | Wald ratio | 1.06(1.03,1.10) | 7.50E-05 | 1.82E-02 |
| LRRC37A2 | Brain Amygdala | Wald ratio | 1.07(1.04,1.10) | 8.93E-06 | 4.34E-03 |
| RPS23 | Brain Amygdala | Wald ratio | 1.08(1.04,1.13) | 2.18E-04 | 3.64E-02 |
| ARL17A | Brain Anterior cingulate corte BA24 | Wald ratio | 1.10(1.05,1.14) | 1.17E-05 | 7.96E-03 |
| CASP8 | Brain Anterior cingulate corte BA24 | Wald ratio | 1.06(1.03,1.09) | 1.25E-04 | 2.84E-02 |
| ENSG00000285668 | Brain Anterior cingulate corte BA24 | Wald ratio | 1.07(1.04,1.10) | 2.30E-05 | 1.05E-02 |
| KANSL1-AS1 | Brain Anterior cingulate corte BA24 | Wald ratio | 1.06(1.03,1.09) | 4.38E-05 | 1.44E-02 |
| LRRC37A2 | Brain Anterior cingulate corte BA24 | Wald ratio | 1.07(1.04,1.10) | 8.93E-06 | 7.96E-03 |
| TDH-AS1 | Brain Anterior cingulate corte BA24 | Wald ratio | 0.90(0.86,0.95) | 5.27E-05 | 1.44E-02 |
| ARL17A | Brain Caudate basal ganglia | Wald ratio | 1.12(1.06,1.17) | 8.88E-06 | 8.99E-03 |
| ENSG00000285668 | Brain Caudate basal ganglia | Wald ratio | 1.07(1.03,1.10) | 2.30E-05 | 1.16E-02 |
| KANSL1-AS1 | Brain Caudate basal ganglia | Wald ratio | 1.07(1.03,1.10) | 2.30E-05 | 1.16E-02 |
| LRRC37A2 | Brain Caudate basal ganglia | Wald ratio | 1.06(1.04,1.09) | 7.38E-06 | 8.99E-03 |
| PLEKHM1 | Brain Caudate basal ganglia | Wald ratio | 1.15(1.07,1.24) | 9.98E-05 | 4.04E-02 |
| ARL17A | Brain Cerebellar Hemisphere | Wald ratio | 1.11(1.06,1.17) | 5.59E-06 | 4.34E-03 |
| CASP8 | Brain Cerebellar Hemisphere | Wald ratio | 1.08(1.03,1.12) | 2.24E-04 | 3.64E-02 |
| ENSG00000285668 | Brain Cerebellar Hemisphere | Wald ratio | 1.07(1.04,1.10) | 2.30E-05 | 7.44E-03 |
| KANSL1-AS1 | Brain Cerebellar Hemisphere | Wald ratio | 1.06(1.03,1.10) | 7.50E-05 | 1.82E-02 |
| LRRC37A2 | Brain Cerebellar Hemisphere | Wald ratio | 1.07(1.04,1.10) | 8.93E-06 | 4.34E-03 |
| RPS23 | Brain Cerebellar Hemisphere | Wald ratio | 1.08(1.04,1.13) | 2.18E-04 | 3.64E-02 |
| ARL17A | Brain Cerebellum | Wald ratio | 1.08(1.04,1.12) | 8.93E-06 | 4.05E-03 |
| ATG10 | Brain Cerebellum | Wald ratio | 0.94(0.91,0.97) | 1.54E-04 | 3.73E-02 |
| CASP8 | Brain Cerebellum | Wald ratio | 1.06(1.03,1.09) | 1.25E-04 | 3.23E-02 |
| ENSG00000236234 | Brain Cerebellum | Wald ratio | 0.86(0.81,0.92) | 6.80E-06 | 4.05E-03 |
| ENSG00000255310 | Brain Cerebellum | Wald ratio | 0.87(0.82,0.93) | 3.99E-05 | 1.21E-02 |
| ENSG00000285668 | Brain Cerebellum | Wald ratio | 1.07(1.03,1.10) | 2.30E-05 | 7.57E-03 |
| FAM215B | Brain Cerebellum | Wald ratio | 1.09(1.05,1.14) | 8.67E-06 | 4.05E-03 |
| FMNL1 | Brain Cerebellum | Wald ratio | 0.90(0.87,0.95) | 7.78E-06 | 4.05E-03 |
| GABBR1 | Brain Cerebellum | Wald ratio | 1.17(1.10,1.25) | 2.23E-06 | 4.05E-03 |
| KANSL1-AS1 | Brain Cerebellum | Wald ratio | 1.06(1.03,1.09) | 2.30E-05 | 7.57E-03 |
| LRRC37A | Brain Cerebellum | Wald ratio | 1.07(1.04,1.10) | 8.93E-06 | 4.05E-03 |
| LRRC37A2 | Brain Cerebellum | Wald ratio | 1.06(1.04,1.09) | 8.22E-06 | 4.05E-03 |
| PLEKHM1 | Brain Cerebellum | Wald ratio | 0.93(0.91,0.96) | 1.41E-05 | 5.67E-03 |
| SPPL2C | Brain Cerebellum | Wald ratio | 1.13(1.07,1.20) | 5.47E-06 | 4.05E-03 |
| TDH-AS1 | Brain Cerebellum | Wald ratio | 0.90(0.85,0.95) | 8.84E-05 | 2.47E-02 |
| ARL17A | Brain Cortex | Wald ratio | 1.07(1.04,1.11) | 1.73E-05 | 1.10E-02 |
| CASP8 | Brain Cortex | Wald ratio | 1.06(1.03,1.09) | 9.05E-05 | 3.11E-02 |
| ENSG00000285668 | Brain Cortex | Wald ratio | 1.06(1.03,1.09) | 2.03E-05 | 1.10E-02 |
| KANSL1-AS1 | Brain Cortex | Wald ratio | 1.06(1.03,1.09) | 2.30E-05 | 1.10E-02 |
| LRRC37A2 | Brain Cortex | Wald ratio | 1.06(1.04,1.09) | 9.94E-06 | 1.10E-02 |
| MAPT-AS1 | Brain Cortex | Wald ratio | 1.21(1.11,1.32) | 1.24E-05 | 1.10E-02 |
| TDH-AS1 | Brain Cortex | Wald ratio | 0.91(0.87,0.95) | 8.85E-05 | 3.11E-02 |
| ARL17A | Brain Frontal Cortex BA9 | Wald ratio | 1.08(1.04,1.11) | 5.15E-06 | 4.83E-03 |
| ATG10 | Brain Frontal Cortex BA9 | Wald ratio | 0.93(0.89,0.96) | 1.35E-04 | 2.98E-02 |
| CASP8 | Brain Frontal Cortex BA9 | Wald ratio | 1.06(1.03,1.08) | 9.05E-05 | 2.92E-02 |
| ENSG00000285668 | Brain Frontal Cortex BA9 | Wald ratio | 1.07(1.04,1.10) | 8.23E-06 | 4.83E-03 |
| ENSG00000285675 | Brain Frontal Cortex BA9 | Wald ratio | 0.91(0.87,0.96) | 1.24E-04 | 2.98E-02 |
| KANSL1-AS1 | Brain Frontal Cortex BA9 | Wald ratio | 1.06(1.03,1.09) | 4.07E-05 | 1.79E-02 |
| LRRC37A2 | Brain Frontal Cortex BA9 | Wald ratio | 1.07(1.04,1.10) | 8.22E-06 | 4.83E-03 |
| TDH-AS1 | Brain Frontal Cortex BA9 | Wald ratio | 0.91(0.87,0.96) | 9.94E-05 | 2.92E-02 |
| ARL17A | Brain Hippocampus | Wald ratio | 1.13(1.07,1.18) | 5.59E-06 | 5.69E-03 |
| CASP8 | Brain Hippocampus | Wald ratio | 1.10(1.04,1.15) | 2.19E-04 | 4.29E-02 |
| ENSG00000285668 | Brain Hippocampus | Wald ratio | 1.07(1.04,1.10) | 2.30E-05 | 9.00E-03 |
| KANSL1-AS1 | Brain Hippocampus | Wald ratio | 1.06(1.03,1.09) | 4.07E-05 | 1.20E-02 |
| LRRC37A2 | Brain Hippocampus | Wald ratio | 1.07(1.04,1.10) | 9.67E-06 | 5.69E-03 |
| TDH-AS1 | Brain Hippocampus | Wald ratio | 0.91(0.87,0.95) | 1.22E-04 | 2.88E-02 |
| ARL17A | Brain Hypothalamus | Wald ratio | 1.09(1.05,1.13) | 2.30E-05 | 7.40E-03 |
| ENSG00000285668 | Brain Hypothalamus | Wald ratio | 1.07(1.04,1.11) | 2.30E-05 | 7.40E-03 |
| FAM215B | Brain Hypothalamus | Wald ratio | 1.12(1.07,1.18) | 5.15E-06 | 5.75E-03 |
| KANSL1-AS1 | Brain Hypothalamus | Wald ratio | 1.06(1.03,1.09) | 4.07E-05 | 1.05E-02 |
| LRRC37A2 | Brain Hypothalamus | Wald ratio | 1.06(1.03,1.09) | 8.93E-06 | 5.75E-03 |
| ARHGAP27 | Brain Nucleus accumbens basal ganglia | Wald ratio | 1.15(1.08,1.22) | 1.73E-05 | 8.50E-03 |
| ARL17A | Brain Nucleus accumbens basal ganglia | Wald ratio | 1.09(1.05,1.13) | 8.93E-06 | 7.23E-03 |
| ENSG00000285668 | Brain Nucleus accumbens basal ganglia | Wald ratio | 1.07(1.04,1.11) | 1.11E-05 | 7.23E-03 |
| FAM215B | Brain Nucleus accumbens basal ganglia | Wald ratio | 1.14(1.07,1.22) | 7.50E-05 | 2.10E-02 |
| KANSL1-AS1 | Brain Nucleus accumbens basal ganglia | Wald ratio | 1.07(1.04,1.10) | 2.30E-05 | 8.55E-03 |
| LRRC37A2 | Brain Nucleus accumbens basal ganglia | Wald ratio | 1.06(1.03,1.09) | 1.11E-05 | 7.23E-03 |
| PLEKHM1 | Brain Nucleus accumbens basal ganglia | Wald ratio | 1.16(1.08,1.24) | 2.62E-05 | 8.55E-03 |
| TDH-AS1 | Brain Nucleus accumbens basal ganglia | Wald ratio | 0.91(0.86,0.95) | 1.42E-04 | 3.47E-02 |
| ARL17A | Brain Putamen basal ganglia | Wald ratio | 1.14(1.07,1.21) | 1.77E-05 | 9.78E-03 |
| CTSB | Brain Putamen basal ganglia | Wald ratio | 1.13(1.06,1.20) | 6.33E-05 | 2.16E-02 |
| ENSG00000285668 | Brain Putamen basal ganglia | Wald ratio | 1.07(1.04,1.10) | 2.30E-05 | 9.78E-03 |
| KANSL1-AS1 | Brain Putamen basal ganglia | Wald ratio | 1.06(1.03,1.10) | 2.30E-05 | 9.78E-03 |
| LRRC37A2 | Brain Putamen basal ganglia | Wald ratio | 1.06(1.03,1.09) | 1.11E-05 | 9.78E-03 |
| ENSG00000265547 | Brain Spinal cord cervical c-1 | Wald ratio | 1.12(1.06,1.17) | 7.09E-06 | 4.59E-03 |
| ENSG00000285668 | Brain Spinal cord cervical c-1 | Wald ratio | 1.06(1.03,1.09) | 2.30E-05 | 4.59E-03 |
| KANSL1-AS1 | Brain Spinal cord cervical c-1 | Wald ratio | 1.06(1.03,1.10) | 2.30E-05 | 4.59E-03 |
| LRRC37A | Brain Spinal cord cervical c-1 | Wald ratio | 1.08(1.04,1.11) | 1.26E-05 | 4.59E-03 |
| LRRC37A2 | Brain Spinal cord cervical c-1 | Wald ratio | 1.07(1.03,1.10) | 2.30E-05 | 4.59E-03 |
| MAPT-AS1 | Brain Spinal cord cervical c-1 | Wald ratio | 0.89(0.85,0.94) | 8.45E-06 | 4.59E-03 |
| KANSL1-AS1 | Brain Substantia nigra | Wald ratio | 1.07(1.04,1.10) | 8.01E-06 | 5.12E-03 |
| LRRC37A | Brain Substantia nigra | Wald ratio | 1.07(1.04,1.10) | 2.30E-05 | 6.73E-03 |
| LRRC37A2 | Brain Substantia nigra | Wald ratio | 1.06(1.04,1.10) | 1.17E-05 | 5.12E-03 |
| TDH-AS1 | Brain Substantia nigra | Wald ratio | 0.92(0.88,0.96) | 8.85E-05 | 1.95E-02 |
| ENSG00000285668 | Whole Blood | Wald ratio | 1.06(1.03,1.09) | 2.30E-05 | 2.76E-02 |
| KANSL1-AS1 | Whole Blood | Wald ratio | 1.06(1.03,1.09) | 4.07E-05 | 3.67E-02 |
| KIZ | Whole Blood | Wald ratio | 1.21(1.12,1.30) | 7.13E-07 | 2.57E-03 |
| LRRC37A | Whole Blood | Wald ratio | 1.12(1.07,1.18) | 8.45E-06 | 1.52E-02 |
