# Supplementary material for: Unveiling promising drug targets for autism spectrum disorder: insights from genetics, transcriptomics, and proteomics
Source: Brief Bioinform. 2024 Jul 22;25(4):bbae353. doi: 10.1093/bib/bbae353 (PMC11262832; doi:10.1093/bib/bbae353)
Supplement: Supplemental_Table_S3_bbae353 [file supplemental_table_s3_bbae353.docx]

**Supplemental Table S3. Results of Mendelian randomization analysis with eQTL and validation GWAS dataset.**

| **Gene** | **Tissue** | **method** | **OR (95CI)** | **Pvalue** | **FDR** |
| --- | --- | --- | --- | --- | --- |
| CASP8 | Brain Amygdala | Wald ratio | 1.07(1.04,1.11) | 7.58E-05 | 1.44E-02 |
| CASP8 | Brain Anterior cingulate corte BA24 | Wald ratio | 1.05(1.03,1.08) | 1.15E-04 | 1.67E-02 |
| KANSL1-AS1 | Brain Anterior cingulate corte BA24 | Wald ratio | 1.07(1.04,1.10) | 1.03E-06 | 2.50E-04 |
| LRRC37A2 | Brain Anterior cingulate corte BA24 | Wald ratio | 1.07(1.04,1.10) | 1.41E-07 | 1.02E-04 |
| TDH-AS1 | Brain Anterior cingulate corte BA24 | Wald ratio | 0.91(0.87,0.95) | 1.45E-05 | 2.63E-03 |
| ENSG00000285668 | Brain Anterior cingulate corte BA24 | Wald ratio | 1.07(1.04,1.10) | 3.14E-07 | 1.14E-04 |
| CTSB | Brain Caudate basal ganglia | Wald ratio | 1.12(1.06,1.18) | 1.23E-04 | 3.78E-02 |
| ARL17A | Brain Caudate basal ganglia | Wald ratio | 1.12(1.07,1.17) | 5.55E-07 | 2.55E-04 |
| KANSL1-AS1 | Brain Caudate basal ganglia | Wald ratio | 1.07(1.04,1.10) | 3.14E-07 | 1.93E-04 |
| PLEKHM1 | Brain Caudate basal ganglia | Wald ratio | 1.16(1.09,1.24) | 3.80E-06 | 1.40E-03 |
| LRRC37A2 | Brain Caudate basal ganglia | Wald ratio | 1.07(1.04,1.09) | 1.09E-07 | 1.93E-04 |
| ENSG00000285668 | Brain Caudate basal ganglia | Wald ratio | 1.07(1.04,1.10) | 3.14E-07 | 1.93E-04 |
| ARL17A | Brain Cerebellar Hemisphere | Wald ratio | 1.12(1.07,1.17) | 1.97E-07 | 6.15E-05 |
| KANSL1-AS1 | Brain Cerebellar Hemisphere | IVW | 1.07(1.05,1.10) | 4.58E-08 | 4.30E-05 |
| LRRC37A2 | Brain Cerebellar Hemisphere | Wald ratio | 1.07(1.04,1.10) | 1.25E-07 | 5.88E-05 |
| ENSG00000285668 | Brain Cerebellar Hemisphere | Wald ratio | 1.08(1.05,1.11) | 3.14E-07 | 7.38E-05 |
| ARHGAP27 | Brain Nucleus accumbens basal ganglia | Wald ratio | 1.15(1.09,1.22) | 8.54E-07 | 3.01E-04 |
| ARL17A | Brain Nucleus accumbens basal ganglia | Wald ratio | 1.10(1.06,1.13) | 1.25E-07 | 1.64E-04 |
| KANSL1-AS1 | Brain Nucleus accumbens basal ganglia | Wald ratio | 1.08(1.05,1.11) | 3.14E-07 | 1.84E-04 |
| PLEKHM1 | Brain Nucleus accumbens basal ganglia | Wald ratio | 1.17(1.10,1.25) | 5.22E-07 | 2.30E-04 |
| FAM215B | Brain Nucleus accumbens basal ganglia | Wald ratio | 1.15(1.08,1.22) | 2.85E-06 | 8.35E-04 |
| LRRC37A2 | Brain Nucleus accumbens basal ganglia | Wald ratio | 1.07(1.04,1.09) | 1.86E-07 | 1.64E-04 |
| TDH-AS1 | Brain Nucleus accumbens basal ganglia | Wald ratio | 0.91(0.87,0.95) | 2.69E-05 | 6.76E-03 |
| ATG10 | Brain Cerebellum | Wald ratio | 0.95(0.92,0.98) | 4.25E-04 | 6.21E-02 |
| LRRC37A | Brain Cerebellum | Wald ratio | 1.07(1.04,1.10) | 1.25E-07 | 8.53E-05 |
| FMNL1 | Brain Cerebellum | Wald ratio | 0.90(0.87,0.94) | 1.91E-07 | 9.76E-05 |
| SPPL2C | Brain Cerebellum | Wald ratio | 1.14(1.09,1.20) | 1.11E-07 | 8.53E-05 |
| ARL17A | Brain Cerebellum | Wald ratio | 1.08(1.05,1.12) | 1.25E-07 | 8.53E-05 |
| GABBR1 | Brain Cerebellum | Wald ratio | 1.13(1.06,1.20) | 6.72E-05 | 1.53E-02 |
| KANSL1-AS1 | Brain Cerebellum | Wald ratio | 1.07(1.04,1.10) | 3.14E-07 | 1.07E-04 |
| PLEKHM1 | Brain Cerebellum | Wald ratio | 0.93(0.91,0.96) | 3.01E-07 | 1.07E-04 |
| CASP8 | Brain Cortex | Wald ratio | 1.05(1.03,1.08) | 7.60E-05 | 2.63E-02 |
| ARL17A | Brain Cortex | Wald ratio | 1.08(1.05,1.11) | 8.54E-07 | 4.43E-04 |
| KANSL1-AS1 | Brain Cortex | Wald ratio | 1.07(1.04,1.10) | 3.14E-07 | 3.26E-04 |
| LRRC37A2 | Brain Cortex | Wald ratio | 1.07(1.04,1.10) | 1.60E-07 | 3.26E-04 |
| TDH-AS1 | Brain Cortex | Wald ratio | 0.91(0.87,0.95) | 1.35E-05 | 5.59E-03 |
| MAPT-AS1 | Brain Cortex | Wald ratio | 1.22(1.13,1.32) | 4.99E-07 | 3.45E-04 |
| ENSG00000285668 | Brain Frontal Cortex BA9 | Wald ratio | 1.08(1.05,1.11) | 1.31E-07 | 2.24E-05 |
| CASP8 | Brain Hippocampus | Wald ratio | 1.09(1.05,1.14) | 7.35E-05 | 9.88E-03 |
| ENSG00000285668 | Brain Hippocampus | Wald ratio | 1.07(1.04,1.10) | 3.14E-07 | 1.27E-04 |
| ENSG00000285668 | Brain Hypothalamus | Wald ratio | 1.08(1.05,1.11) | 3.14E-07 | 1.16E-04 |
| KANSL1-AS1 | Brain Spinal cord cervical c-1 | Wald ratio | 1.07(1.04,1.10) | 3.14E-07 | 4.78E-05 |
| LRRC37A2 | Brain Spinal cord cervical c-1 | Wald ratio | 1.07(1.04,1.10) | 3.14E-07 | 4.78E-05 |
| MAPT-AS1 | Brain Spinal cord cervical c-1 | Wald ratio | 0.88(0.84,0.93) | 1.92E-07 | 4.78E-05 |
| ENSG00000285668 | Brain Spinal cord cervical c-1 | Wald ratio | 1.06(1.04,1.09) | 3.14E-07 | 4.78E-05 |
| KANSL1-AS1 | Whole Blood | Wald ratio | 1.06(1.04,1.09) | 8.50E-07 | 9.87E-04 |
