# Supplementary material for: Unveiling promising drug targets for autism spectrum disorder: insights from genetics, transcriptomics, and proteomics
Source: Brief Bioinform. 2024 Jul 22;25(4):bbae353. doi: 10.1093/bib/bbae353 (PMC11262832; doi:10.1093/bib/bbae353)
Supplement: Supplemental_Table_S4_bbae353 [file supplemental_table_s4_bbae353.docx]

**Supplemental Table S4.** Significant Transcriptome-wide association studies (TWAS) Results for Autism.

| **Gene symbol** | **Tissues** | **Model** | **Model**  **R2** | **Model**  **P-value** | **TWAS**  **Z-score** | **TWAS**  **P-value** | **FDR** |  |
| --- | --- | --- | --- | --- | --- | --- | --- | --- |
| ARHGAP27 | Brain_Caudate_basal_ganglia | top1 | 0.16 | 2.70E-08 | 4.51 | 6.48E-06 | 0.0041 |  |
| ARHGAP27 | Brain_Nucleus_accumbens_basal_ganglia | susie | 0.26 | 1.10E-13 | 4.52 | 6.14E-06 | 0.0042 |  |
| ARL17A | Brain_Anterior_cingulate_cortex_BA24 | susie | 0.33 | 3.10E-13 | 3.95 | 7.74E-05 | 0.0245 |  |
| ARL17A | Brain_Caudate_basal_ganglia | top1 | 0.31 | 9.40E-16 | 4.10 | 4.08E-05 | 0.0163 |  |
| ARL17A | Brain_Cerebellum | enet | 0.56 | 2.00E-35 | 3.77 | 1.63E-04 | 0.0412 |  |
| ARL17A | Brain_Nucleus_accumbens_basal_ganglia | enet | 0.32 | 4.10E-17 | 4.12 | 3.79E-05 | 0.0147 |  |
| ARL17A | Brain_Putamen_basal_ganglia | top1 | 0.22 | 5.00E-10 | 4.10 | 4.15E-05 | 0.0163 |  |
| ARL17A | Brain_Amygdala | susie | 0.23 | 2.20E-08 | 4.06 | 4.81E-05 | 0.0141 |  |
| ARL17A | Brain_Cerebellar_Hemisphere | susie | 0.57 | 2.20E-30 | 3.95 | 7.70E-05 | 0.0226 |  |
| ARL17A | Brain_Hippocampus | lasso | 0.18 | 2.60E-08 | 3.91 | 9.25E-05 | 0.0237 |  |
| ARL17A | Brain_Hypothalamus | susie | 0.41 | 1.00E-19 | 3.70 | 2.18E-04 | 0.0339 |  |
| ARL17A | Brain_Substantia_nigra | top1 | 0.14 | 8.30E-05 | 3.89 | 1.01E-04 | 0.0178 |  |
| ATG10 | Brain_Caudate_basal_ganglia | susie | 0.37 | 3.20E-19 | -3.75 | 1.77E-04 | 0.0410 |  |
| ATG10 | Brain_Cerebellum | lasso | 0.58 | 1.60E-37 | -4.04 | 5.35E-05 | 0.0161 |  |
| ATG10 | Brain_Cerebellar_Hemisphere | susie | 0.47 | 1.40E-23 | -3.91 | 9.32E-05 | 0.0261 |  |
| ATG10 | Brain_Hippocampus | susie | 0.43 | 4.30E-20 | -3.89 | 1.02E-04 | 0.0244 |  |
| ATG10 | Brain_Hypothalamus | susie | 0.43 | 6.00E-21 | -3.72 | 2.01E-04 | 0.0339 |  |
| ATG10 | Brain_Substantia_nigra | susie | 0.43 | 7.10E-14 | -3.60 | 3.21E-04 | 0.0432 |  |
| CASP8 | Brain_Anterior_cingulate_cortex_BA24 | susie | 0.37 | 3.00E-15 | 3.87 | 1.11E-04 | 0.0300 |  |
| CASP8 | Brain_Frontal_Cortex_BA9 | enet | 0.49 | 1.40E-24 | 3.98 | 6.83E-05 | 0.0195 |  |
| CASP8 | Brain_Cerebellar_Hemisphere | susie | 0.40 | 2.30E-19 | 3.87 | 1.10E-04 | 0.0295 |  |
| CASP8 | Brain_Cortex | susie | 0.39 | 1.80E-21 | 3.88 | 1.05E-04 | 0.0396 |  |
| CASP8 | Brain_Hippocampus | top1 | 0.04 | 7.70E-03 | 3.84 | 1.25E-04 | 0.0280 |  |
| CTSB | Brain_Putamen_basal_ganglia | enet | 0.27 | 4.60E-12 | 3.86 | 1.14E-04 | 0.0308 |  |
| CTSB | Brain_Cortex | top1 | 0.08 | 4.70E-05 | 3.98 | 6.83E-05 | 0.0293 |  |
| FAM215B | Brain_Cerebellum | top1 | 0.37 | 1.20E-20 | 4.10 | 4.15E-05 | 0.0144 |  |
| FAM215B | Brain_Cerebellar_Hemisphere | lasso | 0.33 | 1.30E-15 | 4.07 | 4.79E-05 | 0.0174 |  |
| **Gene symbol** | | **Tissues** | **Model** | **Model**  **R2** | **Model**  **P-value** | **TWAS**  **Z-score** | **TWAS**  **P-value** | **FDR** |
| FAM215B | Brain_Cerebellum | top1 | 0.37 | 1.20E-20 | 4.10 | 4.15E-05 | 0.0144 |  |
| KANSL1-AS1 | Brain_Anterior_cingulate_cortex_BA24 | susie | 0.51 | 2.40E-22 | 4.52 | 6.12E-06 | 0.0043 |  |
| KANSL1-AS1 | Brain_Caudate_basal_ganglia | susie | 0.51 | 2.30E-28 | 4.52 | 6.10E-06 | 0.0041 |  |
| KANSL1-AS1 | Brain_Cerebellum | lasso | 0.52 | 4.30E-32 | 4.53 | 5.87E-06 | 0.0043 |  |
| KANSL1-AS1 | Brain_Frontal_Cortex_BA9 | top1 | 0.51 | 3.50E-26 | 4.52 | 6.21E-06 | 0.0048 |  |
| KANSL1-AS1 | Brain_Nucleus_accumbens_basal_ganglia | enet | 0.55 | 5.00E-33 | 4.51 | 6.34E-06 | 0.0042 |  |
| KANSL1-AS1 | Brain_Putamen_basal_ganglia | susie | 0.55 | 6.60E-28 | 4.52 | 6.12E-06 | 0.0040 |  |
| KANSL1-AS1 | Brain_Spinal_cord_cervical_c-1 | susie | 0.39 | 4.90E-14 | 4.52 | 6.17E-06 | 0.0024 |  |
| KANSL1-AS1 | Whole_Blood | top1 | 0.62 | 9.20E-119 | 4.52 | 6.21E-06 | 0.0078 |  |
| KANSL1-AS1 | Brain_Amygdala | lasso | 0.48 | 1.00E-18 | 4.59 | 4.36E-06 | 0.0025 |  |
| KANSL1-AS1 | Brain_Cerebellar_Hemisphere | susie | 0.55 | 6.20E-29 | 4.52 | 6.12E-06 | 0.0037 |  |
| KANSL1-AS1 | Brain_Cortex | top1 | 0.58 | 1.00E-35 | 4.52 | 6.21E-06 | 0.0040 |  |
| KANSL1-AS1 | Brain_Hippocampus | top1 | 0.54 | 1.20E-26 | 4.52 | 6.21E-06 | 0.0032 |  |
| KANSL1-AS1 | Brain_Hypothalamus | susie | 0.44 | 1.50E-21 | 4.52 | 6.12E-06 | 0.0023 |  |
| KANSL1-AS1 | Brain_Substantia_nigra | lasso | 0.47 | 2.00E-15 | 4.60 | 4.22E-06 | 0.0021 |  |
| LRRC37A | Brain_Cerebellar_Hemisphere | enet | 0.65 | 5.80E-37 | 3.99 | 6.68E-05 | 0.0207 |  |
| LRRC37A2 | | Brain_Caudate_basal_ganglia | lasso | 0.59 | 7.30E-35 | 4.06 | 4.97E-05 | 0.0165 |
| LRRC37A2 | Brain_Cerebellum | enet | 0.61 | 1.40E-40 | 3.83 | 1.27E-04 | 0.0332 |  |
| LRRC37A2 | Brain_Frontal_Cortex_BA9 | enet | 0.57 | 3.50E-30 | 3.75 | 1.74E-04 | 0.0467 |  |
| LRRC37A2 | Brain_Nucleus_accumbens_basal_ganglia | enet | 0.59 | 1.10E-36 | 3.99 | 6.58E-05 | 0.0208 |  |
| LRRC37A2 | Brain_Putamen_basal_ganglia | enet | 0.55 | 7.80E-28 | 3.79 | 1.51E-04 | 0.0344 |  |
| LRRC37A2 | Brain_Spinal_cord_cervical_c-1 | lasso | 0.41 | 5.00E-15 | 4.20 | 2.73E-05 | 0.0072 |  |
| LRRC37A2 | Brain_Amygdala | susie | 0.53 | 3.60E-21 | 3.97 | 7.23E-05 | 0.0191 |  |
| LRRC37A2 | Brain_Cerebellar_Hemisphere | susie | 0.62 | 4.90E-35 | 4.02 | 5.74E-05 | 0.0197 |  |
| LRRC37A2 | Brain_Hippocampus | enet | 0.55 | 6.60E-28 | 3.68 | 2.36E-04 | 0.0471 |  |
| LRRC37A2 | Brain_Hypothalamus | susie | 0.61 | 1.00E-33 | 3.85 | 1.19E-04 | 0.0224 |  |
| LRRC37A2 | Brain_Substantia_nigra | top1 | 0.40 | 9.00E-13 | 4.10 | 4.08E-05 | 0.0086 |  |
| MAPT-AS1 | Brain_Cerebellum | top1 | 0.04 | 3.80E-03 | -4.52 | 6.21E-06 | 0.0043 |  |
|  |  |  |  |  |  |  |  |  |
| **Gene symbol** | | **Tissues** | **Model** | **Model**  **R2** | **Model**  **P-value** | **TWAS**  **Z-score** | **TWAS**  **P-value** | **FDR** |
| MAPT-AS1 | Brain_Nucleus_accumbens_basal_ganglia | lasso | 0.35 | 1.90E-18 | -4.41 | 1.02E-05 | 0.0052 |  |
| MAPT-AS1 | Brain_Putamen_basal_ganglia | top1 | 0.11 | 2.00E-05 | -4.51 | 6.47E-06 | 0.0040 |  |
| MAPT-AS1 | Brain_Spinal_cord_cervical_c-1 | susie | 0.19 | 5.80E-07 | -4.52 | 6.16E-06 | 0.0024 |  |
| MAPT-AS1 | Brain_Cerebellar_Hemisphere | enet | 0.13 | 2.80E-06 | -5.27 | 1.35E-07 | 0.0008 |  |
| MAPT-AS1 | Brain_Hippocampus | top1 | 0.06 | 1.10E-03 | -4.52 | 6.21E-06 | 0.0032 |  |
| MAPT-AS1 | Brain_Hypothalamus | susie | 0.28 | 8.70E-13 | -4.52 | 6.15E-06 | 0.0023 |  |
| PLEKHM1 | Brain_Cerebellum | susie | 0.53 | 1.30E-32 | -4.14 | 3.51E-05 | 0.0129 |  |
| PLEKHM1 | Brain_Nucleus_accumbens_basal_ganglia | lasso | 0.22 | 1.40E-11 | 3.84 | 1.23E-04 | 0.0365 |  |
| PLEKHM1 | Brain_Cerebellar_Hemisphere | enet | 0.59 | 9.40E-32 | -4.19 | 2.78E-05 | 0.0114 |  |
| PLEKHM1 | Brain_Hypothalamus | enet | 0.03 | 1.20E-02 | 3.87 | 1.09E-04 | 0.0224 |  |
| SPPL2C | Brain_Cerebellum | top1 | 0.36 | 7.70E-20 | 4.51 | 6.48E-06 | 0.0043 |  |
| SPPL2C | Brain_Cerebellar_Hemisphere | lasso | 0.35 | 3.20E-16 | 4.52 | 6.21E-06 | 0.0037 |  |
| SPPL2C | Brain_Cortex | susie | 0.03 | 6.80E-03 | 4.24 | 2.28E-05 | 0.0124 |  |
| TDH-AS1 | Brain_Anterior_cingulate_cortex_BA24 | top1 | 0.28 | 2.10E-11 | -4.07 | 4.76E-05 | 0.0184 |  |
| TDH-AS1 | Brain_Caudate_basal_ganglia | susie | 0.07 | 3.50E-04 | -3.76 | 1.71E-04 | 0.0410 |  |
| TDH-AS1 | Brain_Cerebellum | top1 | 0.34 | 1.20E-18 | -4.09 | 4.33E-05 | 0.0144 |  |
| TDH-AS1 | Brain_Frontal_Cortex_BA9 | top1 | 0.28 | 9.20E-13 | -4.07 | 4.76E-05 | 0.0181 |  |
| TDH-AS1 | Brain_Nucleus_accumbens_basal_ganglia | top1 | 0.24 | 1.00E-12 | -4.07 | 4.76E-05 | 0.0160 |  |
| TDH-AS1 | Brain_Putamen_basal_ganglia | susie | 0.22 | 3.60E-10 | -3.83 | 1.28E-04 | 0.0308 |  |
| TDH-AS1 | Brain_Spinal_cord_cervical_c-1 | susie | 0.19 | 5.50E-07 | -3.74 | 1.83E-04 | 0.0360 |  |
| TDH-AS1 | Brain_Cortex | susie | 0.27 | 3.90E-14 | -3.97 | 7.25E-05 | 0.0293 |  |
| TDH-AS1 | Brain_Hippocampus | susie | 0.29 | 1.20E-12 | -3.97 | 7.23E-05 | 0.0200 |  |
| TDH-AS1 | Brain_Hypothalamus | susie | 0.14 | 5.70E-07 | -3.86 | 1.12E-04 | 0.0224 |  |
| TDH-AS1 | Brain_Substantia_nigra | susie | 0.24 | 1.20E-07 | -3.80 | 1.43E-04 | 0.0227 |  |
