# Supplementary material for: Unveiling promising drug targets for autism spectrum disorder: insights from genetics, transcriptomics, and proteomics
Source: Brief Bioinform. 2024 Jul 22;25(4):bbae353. doi: 10.1093/bib/bbae353 (PMC11262832; doi:10.1093/bib/bbae353)
Supplement: Supplemental_Table_S5_bbae353 [file supplemental_table_s5_bbae353.docx]

**Supplemental Table S5. Colocalization analysis results for eQTL and PGC ASD GWAS in brain and blood tissues.**

| **Gene** | **Tissue** | **PP.H0.abf** | | **PP.H1.abf** | **PP.H2.abf** | **PP.H3.abf** | **PP.H4.abf** | **Sum**  **PPH3**  **PPH4** |
| --- | --- | --- | --- | --- | --- | --- | --- | --- |
| TDH-AS1 | Brain Anterior cingulate corte BA24 | | 2.65E-04 | 2.82E-04 | 5.48E-02 | 5.73E-02 | 8.87E-01 | 0.944 |
| CASP8 | Brain Anterior cingulate corte BA24 | | 2.61E-12 | 5.93E-12 | 5.83E-02 | 1.32E-01 | 8.10E-01 | 0.942 |
| ENSG00000285668 | Brain Anterior cingulate corte BA24 | | 5.93E-15 | 2.19E-12 | 1.86E-03 | 6.85E-01 | 3.13E-01 | 0.998 |
| LRRC37A2 | Brain Anterior cingulate corte BA24 | | 7.60E-18 | 2.82E-15 | 1.85E-03 | 6.86E-01 | 3.12E-01 | 0.998 |
| ARL17A | Brain Anterior cingulate corte BA24 | | 4.10E-08 | 1.52E-05 | 1.87E-03 | 6.91E-01 | 3.07E-01 | 0.998 |
| KANSL1-AS1 | Brain Anterior cingulate corte BA24 | | 1.02E-16 | 3.81E-14 | 2.07E-03 | 7.72E-01 | 2.26E-01 | 0.998 |
| PLEKHM1 | Brain Caudate basal ganglia | | 1.11E-01 | 1.83E-03 | 5.08E-02 | 0.00E+00 | 8.36E-01 | 0.836 |
| LRRC37A2 | Brain Caudate basal ganglia | | 1.42E-25 | 5.24E-23 | 1.85E-03 | 6.85E-01 | 3.14E-01 | 0.999 |
| ARL17A | Brain Caudate basal ganglia | | 1.06E-08 | 3.89E-06 | 1.86E-03 | 6.86E-01 | 3.12E-01 | 0.998 |
| ENSG00000285668 | Brain Caudate basal ganglia | | 9.45E-20 | 3.48E-17 | 1.91E-03 | 7.02E-01 | 2.97E-01 | 0.999 |
| KANSL1-AS1 | Brain Caudate basal ganglia | | 1.87E-24 | 6.96E-22 | 1.97E-03 | 7.32E-01 | 2.66E-01 | 0.998 |
| CASP8 | Brain Cerebellar Hemisphere | | 6.64E-04 | 1.31E-03 | 3.64E-02 | 7.12E-02 | 8.90E-01 | 0.961 |
| RPS23 | Brain Cerebellar Hemisphere | | 4.81E-07 | 1.79E-07 | 1.17E-01 | 4.29E-02 | 8.40E-01 | 0.883 |
| ARL17A | Brain Cerebellar Hemisphere | | 8.67E-07 | 2.98E-04 | 1.97E-03 | 6.75E-01 | 3.23E-01 | 0.998 |
| LRRC37A2 | Brain Cerebellar Hemisphere | | 3.68E-17 | 1.32E-14 | 1.91E-03 | 6.84E-01 | 3.14E-01 | 0.998 |
| ENSG00000285668 | Brain Cerebellar Hemisphere | | 1.25E-10 | 4.46E-08 | 1.92E-03 | 6.85E-01 | 3.13E-01 | 0.998 |
| KANSL1-AS1 | Brain Cerebellar Hemisphere | | 1.07E-15 | 3.87E-13 | 2.33E-03 | 8.40E-01 | 1.58E-01 | 0.998 |
| GABBR1 | Brain Cerebellum | | 1.39E-05 | 5.23E-04 | 1.29E-03 | 4.76E-02 | 9.51E-01 | 0.999 |
| CASP8 | Brain Cerebellum | | 3.15E-14 | 3.28E-14 | 5.94E-02 | 6.08E-02 | 8.80E-01 | 0.941 |
| ATG10 | Brain Cerebellum | | 5.76E-18 | 2.15E-18 | 8.90E-02 | 3.23E-02 | 8.79E-01 | 0.911 |
| TDH-AS1 | Brain Cerebellum | | 7.01E-11 | 1.50E-10 | 4.77E-02 | 1.02E-01 | 8.51E-01 | 0.953 |
| ENSG00000255310 | Brain Cerebellum | | 1.09E-06 | 6.42E-06 | 5.03E-02 | 2.95E-01 | 6.55E-01 | 0.950 |
| ENSG00000236234 | Brain Cerebellum | | 1.26E-06 | 4.42E-04 | 1.89E-03 | 6.64E-01 | 3.33E-01 | 0.997 |
| LRRC37A | Brain Cerebellum | | 7.97E-28 | 3.00E-25 | 1.79E-03 | 6.72E-01 | 3.27E-01 | 0.999 |
| FMNL1 | Brain Cerebellum | | 7.21E-13 | 2.58E-10 | 1.89E-03 | 6.77E-01 | 3.21E-01 | 0.998 |
| PLEKHM1 | Brain Cerebellum | | 5.58E-28 | 2.08E-25 | 1.84E-03 | 6.85E-01 | 3.13E-01 | 0.998 |
| LRRC37A2 | Brain Cerebellum | | 1.92E-34 | 7.18E-32 | 1.83E-03 | 6.86E-01 | 3.12E-01 | 0.998 |
| FAM215B | Brain Cerebellum | | 4.83E-13 | 1.81E-10 | 1.83E-03 | 6.86E-01 | 3.12E-01 | 0.998 |
| ARL17A | Brain Cerebellum | | 9.11E-19 | 3.41E-16 | 1.83E-03 | 6.86E-01 | 3.12E-01 | 0.998 |
| SPPL2C | Brain Cerebellum | | 1.61E-12 | 6.05E-10 | 1.83E-03 | 6.88E-01 | 3.10E-01 | 0.998 |
| KANSL1-AS1 | Brain Cerebellum | | 4.06E-28 | 1.53E-25 | 1.83E-03 | 6.89E-01 | 3.09E-01 | 0.998 |
| ENSG00000285668 | Brain Cerebellum | | 3.19E-27 | 1.19E-24 | 1.86E-03 | 6.94E-01 | 3.04E-01 | 0.998 |
| MAPT-AS1 | Brain Cortex | | 9.32E-04 | 5.36E-04 | 1.12E-02 | 5.48E-03 | 9.82E-01 | 0.987 |
| TDH-AS1 | Brain Cortex | | 1.61E-06 | 3.15E-06 | 5.56E-02 | 1.08E-01 | 8.36E-01 | 0.944 |
| CASP8 | Brain Cortex | | 2.01E-23 | 4.81E-23 | 5.47E-02 | 1.30E-01 | 8.15E-01 | 0.945 |
| LRRC37A2 | Brain Cortex | | 2.61E-27 | 9.64E-25 | 1.86E-03 | 6.87E-01 | 3.11E-01 | 0.998 |
| ARL17A | Brain Cortex | | 3.30E-15 | 1.22E-12 | 1.88E-03 | 6.93E-01 | 3.05E-01 | 0.998 |
| ENSG00000285668 | Brain Cortex | | 3.18E-21 | 1.17E-18 | 1.89E-03 | 6.93E-01 | 3.05E-01 | 0.998 |
| KANSL1-AS1 | Brain Cortex | | 2.02E-31 | 7.50E-29 | 1.87E-03 | 6.93E-01 | 3.05E-01 | 0.998 |
| LRRC37A2 | Brain Frontal Cortex BA10 | | 8.80E-21 | 3.28E-18 | 1.90E-03 | 7.06E-01 | 2.92E-01 | 0.998 |
| ENSG00000285668 | Brain Frontal Cortex BA11 | | 3.60E-15 | 1.33E-12 | 1.85E-03 | 6.86E-01 | 3.12E-01 | 0.998 |
| KANSL1-AS1 | Brain Frontal Cortex BA12 | | 1.81E-22 | 6.75E-20 | 2.43E-03 | 9.09E-01 | 8.84E-02 | 0.997 |
| CASP8 | Brain Frontal Cortex BA13 | | 1.67E-15 | 4.01E-15 | 5.45E-02 | 1.30E-01 | 8.16E-01 | 0.946 |
| TDH-AS1 | Brain Frontal Cortex BA14 | | 9.25E-06 | 1.79E-05 | 4.95E-02 | 9.49E-02 | 8.56E-01 | 0.951 |
| ENSG00000285675 | Brain Frontal Cortex BA15 | | 2.78E-02 | 1.91E-03 | 8.62E-02 | 5.05E-03 | 8.79E-01 | 0.884 |
| ATG10 | Brain Frontal Cortex BA16 | | 1.09E-08 | 4.04E-09 | 1.22E-01 | 4.44E-02 | 8.34E-01 | 0.878 |
| ARL17A | Brain Frontal Cortex BA9 | | 5.93E-13 | 2.21E-10 | 1.85E-03 | 6.87E-01 | 3.11E-01 | 0.998 |
| CASP8 | Brain Hippocampus | | 2.78E-03 | 8.54E-04 | 6.90E-02 | 2.03E-02 | 9.07E-01 | 0.927 |
| TDH-AS1 | Brain Hippocampus | | 2.04E-05 | 8.48E-05 | 5.29E-02 | 2.18E-01 | 7.29E-01 | 0.947 |
| ARL17A | Brain Hippocampus | | 1.22E-06 | 4.29E-04 | 1.93E-03 | 6.78E-01 | 3.20E-01 | 0.998 |
| LRRC37A2 | Brain Hippocampus | | 2.43E-20 | 8.95E-18 | 1.86E-03 | 6.85E-01 | 3.13E-01 | 0.998 |
| ENSG00000285668 | Brain Hippocampus | | 1.02E-14 | 3.74E-12 | 1.87E-03 | 6.88E-01 | 3.11E-01 | 0.999 |
| KANSL1-AS1 | Brain Hippocampus | | 2.34E-20 | 8.66E-18 | 2.40E-03 | 8.89E-01 | 1.08E-01 | 0.997 |
| FAM215B | Brain Hypothalamus | | 1.06E-06 | 3.67E-04 | 1.95E-03 | 6.75E-01 | 3.22E-01 | 0.997 |
| LRRC37A2 | Brain Hypothalamus | | 4.96E-26 | 1.81E-23 | 1.86E-03 | 6.79E-01 | 3.19E-01 | 0.998 |
| ENSG00000285668 | Brain Hypothalamus | | 8.50E-13 | 3.09E-10 | 1.90E-03 | 6.90E-01 | 3.09E-01 | 0.999 |
| ARL17A | Brain Hypothalamus | | 5.11E-11 | 1.87E-08 | 1.89E-03 | 6.91E-01 | 3.07E-01 | 0.998 |
| KANSL1-AS1 | Brain Hypothalamus | | 4.52E-21 | 1.66E-18 | 2.46E-03 | 9.04E-01 | 9.34E-02 | 0.997 |
| TDH-AS1 | Brain Nucleus accumbens basal ganglia | | 6.42E-02 | 1.25E-03 | 4.56E-02 | 0.00E+00 | 8.89E-01 | 0.889 |
| FAM215B | Brain Nucleus accumbens basal ganglia | | 1.38E-04 | 1.39E-04 | 6.09E-02 | 6.06E-02 | 8.78E-01 | 0.939 |
| ARHGAP27 | Brain Nucleus accumbens basal ganglia | | 1.40E-06 | 4.47E-04 | 2.06E-03 | 6.56E-01 | 3.41E-01 | 0.997 |
| LRRC37A2 | Brain Nucleus accumbens basal ganglia | | 1.09E-30 | 3.99E-28 | 1.86E-03 | 6.84E-01 | 3.14E-01 | 0.998 |
| ENSG00000285668 | Brain Nucleus accumbens basal ganglia | | 8.55E-18 | 3.12E-15 | 1.88E-03 | 6.86E-01 | 3.12E-01 | 0.998 |
| ARL17A | Brain Nucleus accumbens basal ganglia | | 6.39E-12 | 2.35E-09 | 1.87E-03 | 6.87E-01 | 3.11E-01 | 0.998 |
| PLEKHM1 | Brain Nucleus accumbens basal ganglia | | 1.73E-07 | 6.28E-05 | 1.91E-03 | 6.93E-01 | 3.05E-01 | 0.998 |
| KANSL1-AS1 | Brain Nucleus accumbens basal ganglia | | 5.97E-28 | 2.20E-25 | 2.03E-03 | 7.50E-01 | 2.48E-01 | 0.998 |
| CTSB | Brain Putamen basal ganglia | | 5.43E-03 | 7.51E-04 | 9.22E-02 | 1.18E-02 | 8.90E-01 | 0.902 |
| LRRC37A2 | Brain Putamen basal ganglia | | 4.99E-23 | 1.82E-20 | 1.88E-03 | 6.85E-01 | 3.13E-01 | 0.998 |
| ENSG00000285668 | Brain Putamen basal ganglia | | 5.48E-19 | 1.99E-16 | 1.88E-03 | 6.85E-01 | 3.13E-01 | 0.998 |
| ARL17A | Brain Putamen basal ganglia | | 1.90E-07 | 6.84E-05 | 1.90E-03 | 6.87E-01 | 3.11E-01 | 0.998 |
| KANSL1-AS1 | Brain Putamen basal ganglia | | 1.75E-29 | 6.41E-27 | 1.91E-03 | 7.00E-01 | 2.98E-01 | 0.998 |
| MAPT-AS1 | Brain Spinal cord cervical c-1 | | 3.20E-02 | 7.44E-03 | 7.94E-03 | 8.94E-04 | 9.52E-01 | 0.953 |
| ENSG00000265547 | Brain Spinal cord cervical c-1 | | 4.49E-06 | 1.58E-03 | 1.89E-03 | 6.65E-01 | 3.31E-01 | 0.996 |
| LRRC37A2 | Brain Spinal cord cervical c-1 | | 8.99E-12 | 3.42E-09 | 1.80E-03 | 6.84E-01 | 3.14E-01 | 0.998 |
| ENSG00000285668 | Brain Spinal cord cervical c-1 | | 5.52E-13 | 2.10E-10 | 1.81E-03 | 6.85E-01 | 3.13E-01 | 0.998 |
| LRRC37A | Brain Spinal cord cervical c-1 | | 2.83E-10 | 1.08E-07 | 1.80E-03 | 6.86E-01 | 3.12E-01 | 0.998 |
| KANSL1-AS1 | Brain Spinal cord cervical c-1 | | 2.10E-13 | 8.05E-11 | 1.80E-03 | 6.87E-01 | 3.11E-01 | 0.998 |
| TDH-AS1 | Brain Substantia nigra | | 1.84E-03 | 1.31E-03 | 5.48E-02 | 3.79E-02 | 9.04E-01 | 0.942 |
| LRRC37A2 | Brain Substantia nigra | | 3.53E-12 | 1.37E-09 | 1.77E-03 | 6.85E-01 | 3.13E-01 | 0.998 |
| KANSL1-AS1 | Brain Substantia nigra | | 5.90E-11 | 2.30E-08 | 1.76E-03 | 6.87E-01 | 3.11E-01 | 0.998 |
| LRRC37A | Brain Substantia nigra | | 1.20E-09 | 4.67E-07 | 1.77E-03 | 6.87E-01 | 3.11E-01 | 0.998 |
| CASP8 | Brain_Amygdala | | 6.64E-04 | 1.31E-03 | 3.64E-02 | 7.12E-02 | 8.90E-01 | 0.961 |
| RPS23 | Brain_Amygdala | | 4.81E-07 | 1.79E-07 | 1.17E-01 | 4.29E-02 | 8.40E-01 | 0.883 |
| ARL17A | Brain_Amygdala | | 8.67E-07 | 2.98E-04 | 1.97E-03 | 6.75E-01 | 3.23E-01 | 0.998 |
| LRRC37A2 | Brain_Amygdala | | 3.68E-17 | 1.32E-14 | 1.91E-03 | 6.84E-01 | 3.14E-01 | 0.998 |
| ENSG00000285668 | Brain_Amygdala | | 1.25E-10 | 4.46E-08 | 1.92E-03 | 6.85E-01 | 3.13E-01 | 0.998 |
| KANSL1-AS1 | Brain_Amygdala | | 1.07E-15 | 3.87E-13 | 2.33E-03 | 8.40E-01 | 1.58E-01 | 0.998 |
| KIZ | Whole Blood | | 3.76E-21 | 1.91E-18 | 1.05E-03 | 5.33E-01 | 4.66E-01 | 0.999 |
| ENSG00000285668 | Whole Blood | | 2.28E-19 | 4.37E-16 | 2.92E-04 | 5.59E-01 | 4.41E-01 | 1.000 |
| LRRC37A | Whole Blood | | 2.78E-83 | 1.42E-80 | 1.30E-03 | 6.63E-01 | 3.35E-01 | 0.998 |
| KANSL1-AS1 | Whole Blood | | 1.13E-77 | 5.71E-75 | 1.63E-03 | 8.22E-01 | 1.76E-01 | 0.998 |
