# Supplementary material for: Unveiling promising drug targets for autism spectrum disorder: insights from genetics, transcriptomics, and proteomics
Source: Brief Bioinform. 2024 Jul 22;25(4):bbae353. doi: 10.1093/bib/bbae353 (PMC11262832; doi:10.1093/bib/bbae353)
Supplement: Supplemental_Table_S6_bbae353 [file supplemental_table_s6_bbae353.docx]

**Supplemental Table S6.** Significant Summary-data-based Mendelian Randomization (SMR) Associations for Autism.

| Gene | topSNP | beta_SMR | se_SMR | pvalue_SMR | pvalue_HEIDI | nsnp_HEIDI |
| --- | --- | --- | --- | --- | --- | --- |
| ARHGAP27 | rs111423688 | 0.181 | 0.060 | 2.69E-03 | 0.16 | 14 |
| ARL17A | rs2532424 | 0.082 | 0.021 | 1.11E-04 | 0.48 | 12 |
| ATG10 | rs112355050 | -0.086 | 0.024 | 2.94E-04 | 0.15 | 20 |
| CASP8 | rs10200279 | 0.069 | 0.019 | 1.95E-04 | 0.28 | 20 |
| CTSB | rs1122182 | 0.123 | 0.041 | 2.93E-03 | 0.64 | 20 |
| FAM215B | rs199535 | 0.086 | 0.023 | 2.41E-04 | 0.86 | 10 |
| FMNL1 | rs62062283 | -0.113 | 0.028 | 6.35E-05 | NA | NA |
| GABBR1 | rs3025626 | 0.195 | 0.048 | 4.10E-05 | 0.55 | 8 |
| KANSL1-AS1 | rs199535 | 0.067 | 0.018 | 1.19E-04 | 0.32 | 11 |
| LRRC37A2 | rs2942166 | 0.064 | 0.015 | 3.60E-05 | 0.72 | 8 |
| MAPT-AS1 | rs369332489 | -0.114 | 0.033 | 4.87E-04 | 0.54 | 15 |
| PLEKHM1 | rs62065444 | 0.173 | 0.052 | 9.67E-04 | 0.10 | 16 |
| SPPL2C | rs2243967 | 0.112 | 0.028 | 4.98E-05 | 0.70 | 8 |
