# Supplementary material for: Unveiling promising drug targets for autism spectrum disorder: insights from genetics, transcriptomics, and proteomics
Source: Brief Bioinform. 2024 Jul 22;25(4):bbae353. doi: 10.1093/bib/bbae353 (PMC11262832; doi:10.1093/bib/bbae353)
Supplement: Supplemental_Table_S7_bbae353 [file supplemental_table_s7_bbae353.docx]

**Supplemental Table S7.** Phenotypes associated with candidate targets of ASD in knockout mice model.

| ***Gene*** | ***Model*** | ***Genetic Category*** | ***Genotype ID*** | ***Specific Nervous System Phenotype*** | ***Other Abnormal Phenotypes*** | ***Reference***  ***(PMID)*** | |  |
| --- | --- | --- | --- | --- | --- | --- | --- | --- |
| ARHGAP27 |  | Targeted (Conditional ready, Null/knockout, Reporter) (Cell Line) | MGI:4419662 | |  |  | |  |
| ARL17A | No characterized mouse model available |  |  |  |  |  | |  |
| ATG10 |  | Targeted (Null/knockout, Reporter) | MGI:4452822 | |  |  | |  |
| CASP8 | Homozygous knock-out, non-conditional | Targeted (Null/knockout) | MGI:5546196, MGI:3609638 | wavy neural tube, abnormal neural tube morphology, kinked neural tube | cardiovascular, embryo, mortality/aging, cellular, hematopoietic, homeostasis, muscle, | 12654726,  12404118 | |  |
| CTSB | Homo and Heterozygous knock-out, non-conditional | Targeted (Null/knockout) | MGI:2182129 | | cellular, digestive/alimentary, endocrine/exocrine, homeostasis, immune | 9539769 | |  |
| FAM215B | No characterized mouse model available |  |  |  |  |  | |  |
| FMNL1 | conditional knock-out | Targeted (Null/knockout) | MGI:6119535 | | cardiovascular, cellular, hematopoietic, immune, liver/biliary | [28348104](https://www.ncbi.nlm.nih.gov/entrez/query.fcgi?cmd=Retrieve&db=PubMed&list_uids=28348104&dopt=Abstract) | |  |
| GABBR1 | Homo and Heterozygous knock-out, non-conditional | Targeted (Null/knockout) | MGI:2159352, MGI:2159354, MGI:3512742, MGI:3665432, MGI:3665433 | audiogenic seizures, tonic-clonic seizures, sporadic seizures, abnormal axon morphology, abnormal somatic nervous system morphology, abnormal myelination, abnormal nervous system electrophysiology, abnormal synaptic transmission, abnormal inhibitory postsynaptic potential, increased prepulse inhibition, abnormal neuron physiology, abnormal CNS synaptic transmission, abnormal excitatory postsynaptic currents, reduced long-term potentiation | behavior, homeostasis, nervous system, reproductive, growth/size/body, mortality/aging | 11498050,  11414794,  15493018,  16701209,  16701209 |  |  |
| KANSL1-AS1 | No characterized mouse model available |  |  |  |  |  | |  |
| LRRC37A |  | Endonuclease-mediated (Null/knockout) | MGI:7519113 | |  |  | |  |
| LRRC37A2 | No characterized mouse model available |  |  |  |  |  | |  |
| MAPT-AS1 | No characterized mouse model available |  |  |  |  |  | |  |
| PLEKHM1 | Homozygous knock-out, non-conditional | Targeted (Null/knockout, Reporter) | MGI:5513810 | abnormal optic disk morphology, decreased prepulse inhibition | adipose, growth/size/body, homeostasis, limbs/digits/tail, renal/urinary, skeleton, vision/eye |  | |  |
| SPPL2C | Homozygous knock-out, non-conditional | Targeted (Null/knockout) | MGI:6358787 | | cellular, endocrine/exocrine, reproductive | [30733280](https://www.ncbi.nlm.nih.gov/entrez/query.fcgi?cmd=Retrieve&db=PubMed&list_uids=30733280&dopt=Abstract) | |  |
| TDH-AS1 | No characterized mouse model available |  |  |  |  |  | |  |
|  |  |  |  |  |  |  | |  |
