# Supplementary material for: Unveiling promising drug targets for autism spectrum disorder: insights from genetics, transcriptomics, and proteomics
Source: Brief Bioinform. 2024 Jul 22;25(4):bbae353. doi: 10.1093/bib/bbae353 (PMC11262832; doi:10.1093/bib/bbae353)

**Supplemental Information**

**Supplemental Table S1**. Validation of 17 ASD targets in multiple methods.

**Supplemental Table S2**. Estimated casual effects of eQTL on PGC ASD GWAS dataset using Mendelian Randomization.

**Supplemental Table S3.** Results of Mendelian randomization analysis with eQTL and validation GWAS dataset.

**Supplemental Table S4.** Significant Transcriptome-wide association studies (TWAS) Results for Autism.

**Supplemental Table S5.** Colocalization analysis results for eQTL and PGC ASD GWAS in brain and blood tissues.

**Supplemental Table S6.** Significant Summary-data-based Mendelian Randomization (SMR) Associations for Autism.

**Supplemental Table S7.** Phenotypes associated with candidate targets of ASD in knockout mice model.

**Supplementary Figure S1.** Supplementary Figure S1. KEGG pathway enrichment analysis of ASD-risk targets with 4 enriched pathways. Purple represents ATG10, yellow represents CASP8, red represents CTSB and blue represents PLEKHM1.

**Supplementary Figure S2.** GO Cellular Component enrichment analysis of ASD-risk targets with 22 enriched terms. Purple represents ATG10, yellow represents CASP8, red represents CTSB, cyan represents FAM215B, green represents GABBR1, blue represents PLEKHM1 and orange represents SPPL2C.

**Supplementary Figure S3.** GO Molecular Function enrichment analysis of ASD-risk targets with 22 enriched terms. Dark blue represents ARHGAP27, purple represents ATG10, yellow represents CASP8, red represents CTSB, cyan represents FAM215B, green represents GABBR1and orange represents SPPL2C.

**Supplemental Table S1**. Validation of 17 ASD targets in multiple methods.

| **Gene** | **MR-eQTL** | **MR-pQTL** | **Coloc** | **TWAS** | **SMR** |
| --- | --- | --- | --- | --- | --- |
| ARHGAP27 | **√** |  | **√** | **√** | **√** |
| ARL17A | **√** |  | **√** | **√** | **√** |
| ATG10 | **√** |  | **√** | **√** | **√** |
| CASP8 | **√** |  | **√** | **√** | **√** |
| **CTSB** | **√** | **√** | **√** | **√** | **√** |
| FAM215B | **√** |  | **√** | **√** | **√** |
| FMNL1 | **√** | **√** | **√** |  | **√** |
| GABBR1 | **√** | **√** | **√** |  | **√** |
| KANSL1-AS1 | **√** |  | **√** | **√** | **√** |
| LRRC37A | **√** |  | **√** | **√** |  |
| LRRC37A2 | **√** |  | **√** | **√** | **√** |
| MAPT-AS1 | **√** |  | **√** | **√** | **√** |
| PLEKHM1 | **√** |  | **√** | **√** | **√** |
| SPPL2C | **√** |  | **√** | **√** | **√** |
| TDH-AS1 | **√** |  | **√** | **√** |  |
| ENSG00000285675 | **√** |  | **√** |  |  |
| ENSG00000285668 | **√** |  | **√** |  |  |

MR, Mendelian Randomization; eQTL, expression quantitative trait loci; pQTL, protein quantitative trait loci; TWAS: transcriptome-wide association studies; SMR, Summary-data-based Mendelian Randomization.

**Supplemental Table S2. Estimated casual effects of eQTL on PGC ASD GWAS dataset using Mendelian Randomization.**

| **Gene** | **Tissue** | **method** | **OR (95CI)** | **Pvalue** | **FDR** |
| --- | --- | --- | --- | --- | --- |
| ARL17A | Brain Amygdala | Wald ratio | 1.11(1.06,1.17) | 5.59E-06 | 4.34E-03 |
| CASP8 | Brain Amygdala | Wald ratio | 1.08(1.03,1.12) | 2.24E-04 | 3.64E-02 |
| ENSG00000285668 | Brain Amygdala | Wald ratio | 1.07(1.04,1.10) | 2.30E-05 | 7.44E-03 |
| KANSL1-AS1 | Brain Amygdala | Wald ratio | 1.06(1.03,1.10) | 7.50E-05 | 1.82E-02 |
| LRRC37A2 | Brain Amygdala | Wald ratio | 1.07(1.04,1.10) | 8.93E-06 | 4.34E-03 |
| RPS23 | Brain Amygdala | Wald ratio | 1.08(1.04,1.13) | 2.18E-04 | 3.64E-02 |
| ARL17A | Brain Anterior cingulate corte BA24 | Wald ratio | 1.10(1.05,1.14) | 1.17E-05 | 7.96E-03 |
| CASP8 | Brain Anterior cingulate corte BA24 | Wald ratio | 1.06(1.03,1.09) | 1.25E-04 | 2.84E-02 |
| ENSG00000285668 | Brain Anterior cingulate corte BA24 | Wald ratio | 1.07(1.04,1.10) | 2.30E-05 | 1.05E-02 |
| KANSL1-AS1 | Brain Anterior cingulate corte BA24 | Wald ratio | 1.06(1.03,1.09) | 4.38E-05 | 1.44E-02 |
| LRRC37A2 | Brain Anterior cingulate corte BA24 | Wald ratio | 1.07(1.04,1.10) | 8.93E-06 | 7.96E-03 |
| TDH-AS1 | Brain Anterior cingulate corte BA24 | Wald ratio | 0.90(0.86,0.95) | 5.27E-05 | 1.44E-02 |
| ARL17A | Brain Caudate basal ganglia | Wald ratio | 1.12(1.06,1.17) | 8.88E-06 | 8.99E-03 |
| ENSG00000285668 | Brain Caudate basal ganglia | Wald ratio | 1.07(1.03,1.10) | 2.30E-05 | 1.16E-02 |
| KANSL1-AS1 | Brain Caudate basal ganglia | Wald ratio | 1.07(1.03,1.10) | 2.30E-05 | 1.16E-02 |
| LRRC37A2 | Brain Caudate basal ganglia | Wald ratio | 1.06(1.04,1.09) | 7.38E-06 | 8.99E-03 |
| PLEKHM1 | Brain Caudate basal ganglia | Wald ratio | 1.15(1.07,1.24) | 9.98E-05 | 4.04E-02 |
| ARL17A | Brain Cerebellar Hemisphere | Wald ratio | 1.11(1.06,1.17) | 5.59E-06 | 4.34E-03 |
| CASP8 | Brain Cerebellar Hemisphere | Wald ratio | 1.08(1.03,1.12) | 2.24E-04 | 3.64E-02 |
| ENSG00000285668 | Brain Cerebellar Hemisphere | Wald ratio | 1.07(1.04,1.10) | 2.30E-05 | 7.44E-03 |
| KANSL1-AS1 | Brain Cerebellar Hemisphere | Wald ratio | 1.06(1.03,1.10) | 7.50E-05 | 1.82E-02 |
| LRRC37A2 | Brain Cerebellar Hemisphere | Wald ratio | 1.07(1.04,1.10) | 8.93E-06 | 4.34E-03 |
| RPS23 | Brain Cerebellar Hemisphere | Wald ratio | 1.08(1.04,1.13) | 2.18E-04 | 3.64E-02 |
| ARL17A | Brain Cerebellum | Wald ratio | 1.08(1.04,1.12) | 8.93E-06 | 4.05E-03 |
| ATG10 | Brain Cerebellum | Wald ratio | 0.94(0.91,0.97) | 1.54E-04 | 3.73E-02 |
| CASP8 | Brain Cerebellum | Wald ratio | 1.06(1.03,1.09) | 1.25E-04 | 3.23E-02 |
| ENSG00000236234 | Brain Cerebellum | Wald ratio | 0.86(0.81,0.92) | 6.80E-06 | 4.05E-03 |
| ENSG00000255310 | Brain Cerebellum | Wald ratio | 0.87(0.82,0.93) | 3.99E-05 | 1.21E-02 |
| ENSG00000285668 | Brain Cerebellum | Wald ratio | 1.07(1.03,1.10) | 2.30E-05 | 7.57E-03 |
| FAM215B | Brain Cerebellum | Wald ratio | 1.09(1.05,1.14) | 8.67E-06 | 4.05E-03 |
| FMNL1 | Brain Cerebellum | Wald ratio | 0.90(0.87,0.95) | 7.78E-06 | 4.05E-03 |
| GABBR1 | Brain Cerebellum | Wald ratio | 1.17(1.10,1.25) | 2.23E-06 | 4.05E-03 |
| KANSL1-AS1 | Brain Cerebellum | Wald ratio | 1.06(1.03,1.09) | 2.30E-05 | 7.57E-03 |
| LRRC37A | Brain Cerebellum | Wald ratio | 1.07(1.04,1.10) | 8.93E-06 | 4.05E-03 |
| LRRC37A2 | Brain Cerebellum | Wald ratio | 1.06(1.04,1.09) | 8.22E-06 | 4.05E-03 |
| PLEKHM1 | Brain Cerebellum | Wald ratio | 0.93(0.91,0.96) | 1.41E-05 | 5.67E-03 |
| SPPL2C | Brain Cerebellum | Wald ratio | 1.13(1.07,1.20) | 5.47E-06 | 4.05E-03 |
| TDH-AS1 | Brain Cerebellum | Wald ratio | 0.90(0.85,0.95) | 8.84E-05 | 2.47E-02 |
| ARL17A | Brain Cortex | Wald ratio | 1.07(1.04,1.11) | 1.73E-05 | 1.10E-02 |
| CASP8 | Brain Cortex | Wald ratio | 1.06(1.03,1.09) | 9.05E-05 | 3.11E-02 |
| ENSG00000285668 | Brain Cortex | Wald ratio | 1.06(1.03,1.09) | 2.03E-05 | 1.10E-02 |
| KANSL1-AS1 | Brain Cortex | Wald ratio | 1.06(1.03,1.09) | 2.30E-05 | 1.10E-02 |
| LRRC37A2 | Brain Cortex | Wald ratio | 1.06(1.04,1.09) | 9.94E-06 | 1.10E-02 |
| MAPT-AS1 | Brain Cortex | Wald ratio | 1.21(1.11,1.32) | 1.24E-05 | 1.10E-02 |
| TDH-AS1 | Brain Cortex | Wald ratio | 0.91(0.87,0.95) | 8.85E-05 | 3.11E-02 |
| ARL17A | Brain Frontal Cortex BA9 | Wald ratio | 1.08(1.04,1.11) | 5.15E-06 | 4.83E-03 |
| ATG10 | Brain Frontal Cortex BA9 | Wald ratio | 0.93(0.89,0.96) | 1.35E-04 | 2.98E-02 |
| CASP8 | Brain Frontal Cortex BA9 | Wald ratio | 1.06(1.03,1.08) | 9.05E-05 | 2.92E-02 |
| ENSG00000285668 | Brain Frontal Cortex BA9 | Wald ratio | 1.07(1.04,1.10) | 8.23E-06 | 4.83E-03 |
| ENSG00000285675 | Brain Frontal Cortex BA9 | Wald ratio | 0.91(0.87,0.96) | 1.24E-04 | 2.98E-02 |
| KANSL1-AS1 | Brain Frontal Cortex BA9 | Wald ratio | 1.06(1.03,1.09) | 4.07E-05 | 1.79E-02 |
| LRRC37A2 | Brain Frontal Cortex BA9 | Wald ratio | 1.07(1.04,1.10) | 8.22E-06 | 4.83E-03 |
| TDH-AS1 | Brain Frontal Cortex BA9 | Wald ratio | 0.91(0.87,0.96) | 9.94E-05 | 2.92E-02 |
| ARL17A | Brain Hippocampus | Wald ratio | 1.13(1.07,1.18) | 5.59E-06 | 5.69E-03 |
| CASP8 | Brain Hippocampus | Wald ratio | 1.10(1.04,1.15) | 2.19E-04 | 4.29E-02 |
| ENSG00000285668 | Brain Hippocampus | Wald ratio | 1.07(1.04,1.10) | 2.30E-05 | 9.00E-03 |
| KANSL1-AS1 | Brain Hippocampus | Wald ratio | 1.06(1.03,1.09) | 4.07E-05 | 1.20E-02 |
| LRRC37A2 | Brain Hippocampus | Wald ratio | 1.07(1.04,1.10) | 9.67E-06 | 5.69E-03 |
| TDH-AS1 | Brain Hippocampus | Wald ratio | 0.91(0.87,0.95) | 1.22E-04 | 2.88E-02 |
| ARL17A | Brain Hypothalamus | Wald ratio | 1.09(1.05,1.13) | 2.30E-05 | 7.40E-03 |
| ENSG00000285668 | Brain Hypothalamus | Wald ratio | 1.07(1.04,1.11) | 2.30E-05 | 7.40E-03 |
| FAM215B | Brain Hypothalamus | Wald ratio | 1.12(1.07,1.18) | 5.15E-06 | 5.75E-03 |
| KANSL1-AS1 | Brain Hypothalamus | Wald ratio | 1.06(1.03,1.09) | 4.07E-05 | 1.05E-02 |
| LRRC37A2 | Brain Hypothalamus | Wald ratio | 1.06(1.03,1.09) | 8.93E-06 | 5.75E-03 |
| ARHGAP27 | Brain Nucleus accumbens basal ganglia | Wald ratio | 1.15(1.08,1.22) | 1.73E-05 | 8.50E-03 |
| ARL17A | Brain Nucleus accumbens basal ganglia | Wald ratio | 1.09(1.05,1.13) | 8.93E-06 | 7.23E-03 |
| ENSG00000285668 | Brain Nucleus accumbens basal ganglia | Wald ratio | 1.07(1.04,1.11) | 1.11E-05 | 7.23E-03 |
| FAM215B | Brain Nucleus accumbens basal ganglia | Wald ratio | 1.14(1.07,1.22) | 7.50E-05 | 2.10E-02 |
| KANSL1-AS1 | Brain Nucleus accumbens basal ganglia | Wald ratio | 1.07(1.04,1.10) | 2.30E-05 | 8.55E-03 |
| LRRC37A2 | Brain Nucleus accumbens basal ganglia | Wald ratio | 1.06(1.03,1.09) | 1.11E-05 | 7.23E-03 |
| PLEKHM1 | Brain Nucleus accumbens basal ganglia | Wald ratio | 1.16(1.08,1.24) | 2.62E-05 | 8.55E-03 |
| TDH-AS1 | Brain Nucleus accumbens basal ganglia | Wald ratio | 0.91(0.86,0.95) | 1.42E-04 | 3.47E-02 |
| ARL17A | Brain Putamen basal ganglia | Wald ratio | 1.14(1.07,1.21) | 1.77E-05 | 9.78E-03 |
| CTSB | Brain Putamen basal ganglia | Wald ratio | 1.13(1.06,1.20) | 6.33E-05 | 2.16E-02 |
| ENSG00000285668 | Brain Putamen basal ganglia | Wald ratio | 1.07(1.04,1.10) | 2.30E-05 | 9.78E-03 |
| KANSL1-AS1 | Brain Putamen basal ganglia | Wald ratio | 1.06(1.03,1.10) | 2.30E-05 | 9.78E-03 |
| LRRC37A2 | Brain Putamen basal ganglia | Wald ratio | 1.06(1.03,1.09) | 1.11E-05 | 9.78E-03 |
| ENSG00000265547 | Brain Spinal cord cervical c-1 | Wald ratio | 1.12(1.06,1.17) | 7.09E-06 | 4.59E-03 |
| ENSG00000285668 | Brain Spinal cord cervical c-1 | Wald ratio | 1.06(1.03,1.09) | 2.30E-05 | 4.59E-03 |
| KANSL1-AS1 | Brain Spinal cord cervical c-1 | Wald ratio | 1.06(1.03,1.10) | 2.30E-05 | 4.59E-03 |
| LRRC37A | Brain Spinal cord cervical c-1 | Wald ratio | 1.08(1.04,1.11) | 1.26E-05 | 4.59E-03 |
| LRRC37A2 | Brain Spinal cord cervical c-1 | Wald ratio | 1.07(1.03,1.10) | 2.30E-05 | 4.59E-03 |
| MAPT-AS1 | Brain Spinal cord cervical c-1 | Wald ratio | 0.89(0.85,0.94) | 8.45E-06 | 4.59E-03 |
| KANSL1-AS1 | Brain Substantia nigra | Wald ratio | 1.07(1.04,1.10) | 8.01E-06 | 5.12E-03 |
| LRRC37A | Brain Substantia nigra | Wald ratio | 1.07(1.04,1.10) | 2.30E-05 | 6.73E-03 |
| LRRC37A2 | Brain Substantia nigra | Wald ratio | 1.06(1.04,1.10) | 1.17E-05 | 5.12E-03 |
| TDH-AS1 | Brain Substantia nigra | Wald ratio | 0.92(0.88,0.96) | 8.85E-05 | 1.95E-02 |
| ENSG00000285668 | Whole Blood | Wald ratio | 1.06(1.03,1.09) | 2.30E-05 | 2.76E-02 |
| KANSL1-AS1 | Whole Blood | Wald ratio | 1.06(1.03,1.09) | 4.07E-05 | 3.67E-02 |
| KIZ | Whole Blood | Wald ratio | 1.21(1.12,1.30) | 7.13E-07 | 2.57E-03 |
| LRRC37A | Whole Blood | Wald ratio | 1.12(1.07,1.18) | 8.45E-06 | 1.52E-02 |

**Supplemental Table S3. Results of Mendelian randomization analysis with eQTL and validation GWAS dataset.**

| **Gene** | **Tissue** | **method** | **OR (95CI)** | **Pvalue** | **FDR** |
| --- | --- | --- | --- | --- | --- |
| CASP8 | Brain Amygdala | Wald ratio | 1.07(1.04,1.11) | 7.58E-05 | 1.44E-02 |
| CASP8 | Brain Anterior cingulate corte BA24 | Wald ratio | 1.05(1.03,1.08) | 1.15E-04 | 1.67E-02 |
| KANSL1-AS1 | Brain Anterior cingulate corte BA24 | Wald ratio | 1.07(1.04,1.10) | 1.03E-06 | 2.50E-04 |
| LRRC37A2 | Brain Anterior cingulate corte BA24 | Wald ratio | 1.07(1.04,1.10) | 1.41E-07 | 1.02E-04 |
| TDH-AS1 | Brain Anterior cingulate corte BA24 | Wald ratio | 0.91(0.87,0.95) | 1.45E-05 | 2.63E-03 |
| ENSG00000285668 | Brain Anterior cingulate corte BA24 | Wald ratio | 1.07(1.04,1.10) | 3.14E-07 | 1.14E-04 |
| CTSB | Brain Caudate basal ganglia | Wald ratio | 1.12(1.06,1.18) | 1.23E-04 | 3.78E-02 |
| ARL17A | Brain Caudate basal ganglia | Wald ratio | 1.12(1.07,1.17) | 5.55E-07 | 2.55E-04 |
| KANSL1-AS1 | Brain Caudate basal ganglia | Wald ratio | 1.07(1.04,1.10) | 3.14E-07 | 1.93E-04 |
| PLEKHM1 | Brain Caudate basal ganglia | Wald ratio | 1.16(1.09,1.24) | 3.80E-06 | 1.40E-03 |
| LRRC37A2 | Brain Caudate basal ganglia | Wald ratio | 1.07(1.04,1.09) | 1.09E-07 | 1.93E-04 |
| ENSG00000285668 | Brain Caudate basal ganglia | Wald ratio | 1.07(1.04,1.10) | 3.14E-07 | 1.93E-04 |
| ARL17A | Brain Cerebellar Hemisphere | Wald ratio | 1.12(1.07,1.17) | 1.97E-07 | 6.15E-05 |
| KANSL1-AS1 | Brain Cerebellar Hemisphere | IVW | 1.07(1.05,1.10) | 4.58E-08 | 4.30E-05 |
| LRRC37A2 | Brain Cerebellar Hemisphere | Wald ratio | 1.07(1.04,1.10) | 1.25E-07 | 5.88E-05 |
| ENSG00000285668 | Brain Cerebellar Hemisphere | Wald ratio | 1.08(1.05,1.11) | 3.14E-07 | 7.38E-05 |
| ARHGAP27 | Brain Nucleus accumbens basal ganglia | Wald ratio | 1.15(1.09,1.22) | 8.54E-07 | 3.01E-04 |
| ARL17A | Brain Nucleus accumbens basal ganglia | Wald ratio | 1.10(1.06,1.13) | 1.25E-07 | 1.64E-04 |
| KANSL1-AS1 | Brain Nucleus accumbens basal ganglia | Wald ratio | 1.08(1.05,1.11) | 3.14E-07 | 1.84E-04 |
| PLEKHM1 | Brain Nucleus accumbens basal ganglia | Wald ratio | 1.17(1.10,1.25) | 5.22E-07 | 2.30E-04 |
| FAM215B | Brain Nucleus accumbens basal ganglia | Wald ratio | 1.15(1.08,1.22) | 2.85E-06 | 8.35E-04 |
| LRRC37A2 | Brain Nucleus accumbens basal ganglia | Wald ratio | 1.07(1.04,1.09) | 1.86E-07 | 1.64E-04 |
| TDH-AS1 | Brain Nucleus accumbens basal ganglia | Wald ratio | 0.91(0.87,0.95) | 2.69E-05 | 6.76E-03 |
| ATG10 | Brain Cerebellum | Wald ratio | 0.95(0.92,0.98) | 4.25E-04 | 6.21E-02 |
| LRRC37A | Brain Cerebellum | Wald ratio | 1.07(1.04,1.10) | 1.25E-07 | 8.53E-05 |
| FMNL1 | Brain Cerebellum | Wald ratio | 0.90(0.87,0.94) | 1.91E-07 | 9.76E-05 |
| SPPL2C | Brain Cerebellum | Wald ratio | 1.14(1.09,1.20) | 1.11E-07 | 8.53E-05 |
| ARL17A | Brain Cerebellum | Wald ratio | 1.08(1.05,1.12) | 1.25E-07 | 8.53E-05 |
| GABBR1 | Brain Cerebellum | Wald ratio | 1.13(1.06,1.20) | 6.72E-05 | 1.53E-02 |
| KANSL1-AS1 | Brain Cerebellum | Wald ratio | 1.07(1.04,1.10) | 3.14E-07 | 1.07E-04 |
| PLEKHM1 | Brain Cerebellum | Wald ratio | 0.93(0.91,0.96) | 3.01E-07 | 1.07E-04 |
| CASP8 | Brain Cortex | Wald ratio | 1.05(1.03,1.08) | 7.60E-05 | 2.63E-02 |
| ARL17A | Brain Cortex | Wald ratio | 1.08(1.05,1.11) | 8.54E-07 | 4.43E-04 |
| KANSL1-AS1 | Brain Cortex | Wald ratio | 1.07(1.04,1.10) | 3.14E-07 | 3.26E-04 |
| LRRC37A2 | Brain Cortex | Wald ratio | 1.07(1.04,1.10) | 1.60E-07 | 3.26E-04 |
| TDH-AS1 | Brain Cortex | Wald ratio | 0.91(0.87,0.95) | 1.35E-05 | 5.59E-03 |
| MAPT-AS1 | Brain Cortex | Wald ratio | 1.22(1.13,1.32) | 4.99E-07 | 3.45E-04 |
| ENSG00000285668 | Brain Frontal Cortex BA9 | Wald ratio | 1.08(1.05,1.11) | 1.31E-07 | 2.24E-05 |
| CASP8 | Brain Hippocampus | Wald ratio | 1.09(1.05,1.14) | 7.35E-05 | 9.88E-03 |
| ENSG00000285668 | Brain Hippocampus | Wald ratio | 1.07(1.04,1.10) | 3.14E-07 | 1.27E-04 |
| ENSG00000285668 | Brain Hypothalamus | Wald ratio | 1.08(1.05,1.11) | 3.14E-07 | 1.16E-04 |
| KANSL1-AS1 | Brain Spinal cord cervical c-1 | Wald ratio | 1.07(1.04,1.10) | 3.14E-07 | 4.78E-05 |
| LRRC37A2 | Brain Spinal cord cervical c-1 | Wald ratio | 1.07(1.04,1.10) | 3.14E-07 | 4.78E-05 |
| MAPT-AS1 | Brain Spinal cord cervical c-1 | Wald ratio | 0.88(0.84,0.93) | 1.92E-07 | 4.78E-05 |
| ENSG00000285668 | Brain Spinal cord cervical c-1 | Wald ratio | 1.06(1.04,1.09) | 3.14E-07 | 4.78E-05 |
| KANSL1-AS1 | Whole Blood | Wald ratio | 1.06(1.04,1.09) | 8.50E-07 | 9.87E-04 |

**Supplemental Table S4.** Significant Transcriptome-wide association studies (TWAS) Results for Autism.

| **Gene symbol** | **Tissues** | **Model** | **Model**  **R2** | **Model**  **P-value** | **TWAS**  **Z-score** | **TWAS**  **P-value** | **FDR** |  |
| --- | --- | --- | --- | --- | --- | --- | --- | --- |
| ARHGAP27 | Brain_Caudate_basal_ganglia | top1 | 0.16 | 2.70E-08 | 4.51 | 6.48E-06 | 0.0041 |  |
| ARHGAP27 | Brain_Nucleus_accumbens_basal_ganglia | susie | 0.26 | 1.10E-13 | 4.52 | 6.14E-06 | 0.0042 |  |
| ARL17A | Brain_Anterior_cingulate_cortex_BA24 | susie | 0.33 | 3.10E-13 | 3.95 | 7.74E-05 | 0.0245 |  |
| ARL17A | Brain_Caudate_basal_ganglia | top1 | 0.31 | 9.40E-16 | 4.10 | 4.08E-05 | 0.0163 |  |
| ARL17A | Brain_Cerebellum | enet | 0.56 | 2.00E-35 | 3.77 | 1.63E-04 | 0.0412 |  |
| ARL17A | Brain_Nucleus_accumbens_basal_ganglia | enet | 0.32 | 4.10E-17 | 4.12 | 3.79E-05 | 0.0147 |  |
| ARL17A | Brain_Putamen_basal_ganglia | top1 | 0.22 | 5.00E-10 | 4.10 | 4.15E-05 | 0.0163 |  |
| ARL17A | Brain_Amygdala | susie | 0.23 | 2.20E-08 | 4.06 | 4.81E-05 | 0.0141 |  |
| ARL17A | Brain_Cerebellar_Hemisphere | susie | 0.57 | 2.20E-30 | 3.95 | 7.70E-05 | 0.0226 |  |
| ARL17A | Brain_Hippocampus | lasso | 0.18 | 2.60E-08 | 3.91 | 9.25E-05 | 0.0237 |  |
| ARL17A | Brain_Hypothalamus | susie | 0.41 | 1.00E-19 | 3.70 | 2.18E-04 | 0.0339 |  |
| ARL17A | Brain_Substantia_nigra | top1 | 0.14 | 8.30E-05 | 3.89 | 1.01E-04 | 0.0178 |  |
| ATG10 | Brain_Caudate_basal_ganglia | susie | 0.37 | 3.20E-19 | -3.75 | 1.77E-04 | 0.0410 |  |
| ATG10 | Brain_Cerebellum | lasso | 0.58 | 1.60E-37 | -4.04 | 5.35E-05 | 0.0161 |  |
| ATG10 | Brain_Cerebellar_Hemisphere | susie | 0.47 | 1.40E-23 | -3.91 | 9.32E-05 | 0.0261 |  |
| ATG10 | Brain_Hippocampus | susie | 0.43 | 4.30E-20 | -3.89 | 1.02E-04 | 0.0244 |  |
| ATG10 | Brain_Hypothalamus | susie | 0.43 | 6.00E-21 | -3.72 | 2.01E-04 | 0.0339 |  |
| ATG10 | Brain_Substantia_nigra | susie | 0.43 | 7.10E-14 | -3.60 | 3.21E-04 | 0.0432 |  |
| CASP8 | Brain_Anterior_cingulate_cortex_BA24 | susie | 0.37 | 3.00E-15 | 3.87 | 1.11E-04 | 0.0300 |  |
| CASP8 | Brain_Frontal_Cortex_BA9 | enet | 0.49 | 1.40E-24 | 3.98 | 6.83E-05 | 0.0195 |  |
| CASP8 | Brain_Cerebellar_Hemisphere | susie | 0.40 | 2.30E-19 | 3.87 | 1.10E-04 | 0.0295 |  |
| CASP8 | Brain_Cortex | susie | 0.39 | 1.80E-21 | 3.88 | 1.05E-04 | 0.0396 |  |
| CASP8 | Brain_Hippocampus | top1 | 0.04 | 7.70E-03 | 3.84 | 1.25E-04 | 0.0280 |  |
| CTSB | Brain_Putamen_basal_ganglia | enet | 0.27 | 4.60E-12 | 3.86 | 1.14E-04 | 0.0308 |  |
| CTSB | Brain_Cortex | top1 | 0.08 | 4.70E-05 | 3.98 | 6.83E-05 | 0.0293 |  |
| FAM215B | Brain_Cerebellum | top1 | 0.37 | 1.20E-20 | 4.10 | 4.15E-05 | 0.0144 |  |
| FAM215B | Brain_Cerebellar_Hemisphere | lasso | 0.33 | 1.30E-15 | 4.07 | 4.79E-05 | 0.0174 |  |
| **Gene symbol** | | **Tissues** | **Model** | **Model**  **R2** | **Model**  **P-value** | **TWAS**  **Z-score** | **TWAS**  **P-value** | **FDR** |
| FAM215B | Brain_Cerebellum | top1 | 0.37 | 1.20E-20 | 4.10 | 4.15E-05 | 0.0144 |  |
| KANSL1-AS1 | Brain_Anterior_cingulate_cortex_BA24 | susie | 0.51 | 2.40E-22 | 4.52 | 6.12E-06 | 0.0043 |  |
| KANSL1-AS1 | Brain_Caudate_basal_ganglia | susie | 0.51 | 2.30E-28 | 4.52 | 6.10E-06 | 0.0041 |  |
| KANSL1-AS1 | Brain_Cerebellum | lasso | 0.52 | 4.30E-32 | 4.53 | 5.87E-06 | 0.0043 |  |
| KANSL1-AS1 | Brain_Frontal_Cortex_BA9 | top1 | 0.51 | 3.50E-26 | 4.52 | 6.21E-06 | 0.0048 |  |
| KANSL1-AS1 | Brain_Nucleus_accumbens_basal_ganglia | enet | 0.55 | 5.00E-33 | 4.51 | 6.34E-06 | 0.0042 |  |
| KANSL1-AS1 | Brain_Putamen_basal_ganglia | susie | 0.55 | 6.60E-28 | 4.52 | 6.12E-06 | 0.0040 |  |
| KANSL1-AS1 | Brain_Spinal_cord_cervical_c-1 | susie | 0.39 | 4.90E-14 | 4.52 | 6.17E-06 | 0.0024 |  |
| KANSL1-AS1 | Whole_Blood | top1 | 0.62 | 9.20E-119 | 4.52 | 6.21E-06 | 0.0078 |  |
| KANSL1-AS1 | Brain_Amygdala | lasso | 0.48 | 1.00E-18 | 4.59 | 4.36E-06 | 0.0025 |  |
| KANSL1-AS1 | Brain_Cerebellar_Hemisphere | susie | 0.55 | 6.20E-29 | 4.52 | 6.12E-06 | 0.0037 |  |
| KANSL1-AS1 | Brain_Cortex | top1 | 0.58 | 1.00E-35 | 4.52 | 6.21E-06 | 0.0040 |  |
| KANSL1-AS1 | Brain_Hippocampus | top1 | 0.54 | 1.20E-26 | 4.52 | 6.21E-06 | 0.0032 |  |
| KANSL1-AS1 | Brain_Hypothalamus | susie | 0.44 | 1.50E-21 | 4.52 | 6.12E-06 | 0.0023 |  |
| KANSL1-AS1 | Brain_Substantia_nigra | lasso | 0.47 | 2.00E-15 | 4.60 | 4.22E-06 | 0.0021 |  |
| LRRC37A | Brain_Cerebellar_Hemisphere | enet | 0.65 | 5.80E-37 | 3.99 | 6.68E-05 | 0.0207 |  |
| LRRC37A2 | | Brain_Caudate_basal_ganglia | lasso | 0.59 | 7.30E-35 | 4.06 | 4.97E-05 | 0.0165 |
| LRRC37A2 | Brain_Cerebellum | enet | 0.61 | 1.40E-40 | 3.83 | 1.27E-04 | 0.0332 |  |
| LRRC37A2 | Brain_Frontal_Cortex_BA9 | enet | 0.57 | 3.50E-30 | 3.75 | 1.74E-04 | 0.0467 |  |
| LRRC37A2 | Brain_Nucleus_accumbens_basal_ganglia | enet | 0.59 | 1.10E-36 | 3.99 | 6.58E-05 | 0.0208 |  |
| LRRC37A2 | Brain_Putamen_basal_ganglia | enet | 0.55 | 7.80E-28 | 3.79 | 1.51E-04 | 0.0344 |  |
| LRRC37A2 | Brain_Spinal_cord_cervical_c-1 | lasso | 0.41 | 5.00E-15 | 4.20 | 2.73E-05 | 0.0072 |  |
| LRRC37A2 | Brain_Amygdala | susie | 0.53 | 3.60E-21 | 3.97 | 7.23E-05 | 0.0191 |  |
| LRRC37A2 | Brain_Cerebellar_Hemisphere | susie | 0.62 | 4.90E-35 | 4.02 | 5.74E-05 | 0.0197 |  |
| LRRC37A2 | Brain_Hippocampus | enet | 0.55 | 6.60E-28 | 3.68 | 2.36E-04 | 0.0471 |  |
| LRRC37A2 | Brain_Hypothalamus | susie | 0.61 | 1.00E-33 | 3.85 | 1.19E-04 | 0.0224 |  |
| LRRC37A2 | Brain_Substantia_nigra | top1 | 0.40 | 9.00E-13 | 4.10 | 4.08E-05 | 0.0086 |  |
| MAPT-AS1 | Brain_Cerebellum | top1 | 0.04 | 3.80E-03 | -4.52 | 6.21E-06 | 0.0043 |  |
|  |  |  |  |  |  |  |  |  |
| **Gene symbol** | | **Tissues** | **Model** | **Model**  **R2** | **Model**  **P-value** | **TWAS**  **Z-score** | **TWAS**  **P-value** | **FDR** |
| MAPT-AS1 | Brain_Nucleus_accumbens_basal_ganglia | lasso | 0.35 | 1.90E-18 | -4.41 | 1.02E-05 | 0.0052 |  |
| MAPT-AS1 | Brain_Putamen_basal_ganglia | top1 | 0.11 | 2.00E-05 | -4.51 | 6.47E-06 | 0.0040 |  |
| MAPT-AS1 | Brain_Spinal_cord_cervical_c-1 | susie | 0.19 | 5.80E-07 | -4.52 | 6.16E-06 | 0.0024 |  |
| MAPT-AS1 | Brain_Cerebellar_Hemisphere | enet | 0.13 | 2.80E-06 | -5.27 | 1.35E-07 | 0.0008 |  |
| MAPT-AS1 | Brain_Hippocampus | top1 | 0.06 | 1.10E-03 | -4.52 | 6.21E-06 | 0.0032 |  |
| MAPT-AS1 | Brain_Hypothalamus | susie | 0.28 | 8.70E-13 | -4.52 | 6.15E-06 | 0.0023 |  |
| PLEKHM1 | Brain_Cerebellum | susie | 0.53 | 1.30E-32 | -4.14 | 3.51E-05 | 0.0129 |  |
| PLEKHM1 | Brain_Nucleus_accumbens_basal_ganglia | lasso | 0.22 | 1.40E-11 | 3.84 | 1.23E-04 | 0.0365 |  |
| PLEKHM1 | Brain_Cerebellar_Hemisphere | enet | 0.59 | 9.40E-32 | -4.19 | 2.78E-05 | 0.0114 |  |
| PLEKHM1 | Brain_Hypothalamus | enet | 0.03 | 1.20E-02 | 3.87 | 1.09E-04 | 0.0224 |  |
| SPPL2C | Brain_Cerebellum | top1 | 0.36 | 7.70E-20 | 4.51 | 6.48E-06 | 0.0043 |  |
| SPPL2C | Brain_Cerebellar_Hemisphere | lasso | 0.35 | 3.20E-16 | 4.52 | 6.21E-06 | 0.0037 |  |
| SPPL2C | Brain_Cortex | susie | 0.03 | 6.80E-03 | 4.24 | 2.28E-05 | 0.0124 |  |
| TDH-AS1 | Brain_Anterior_cingulate_cortex_BA24 | top1 | 0.28 | 2.10E-11 | -4.07 | 4.76E-05 | 0.0184 |  |
| TDH-AS1 | Brain_Caudate_basal_ganglia | susie | 0.07 | 3.50E-04 | -3.76 | 1.71E-04 | 0.0410 |  |
| TDH-AS1 | Brain_Cerebellum | top1 | 0.34 | 1.20E-18 | -4.09 | 4.33E-05 | 0.0144 |  |
| TDH-AS1 | Brain_Frontal_Cortex_BA9 | top1 | 0.28 | 9.20E-13 | -4.07 | 4.76E-05 | 0.0181 |  |
| TDH-AS1 | Brain_Nucleus_accumbens_basal_ganglia | top1 | 0.24 | 1.00E-12 | -4.07 | 4.76E-05 | 0.0160 |  |
| TDH-AS1 | Brain_Putamen_basal_ganglia | susie | 0.22 | 3.60E-10 | -3.83 | 1.28E-04 | 0.0308 |  |
| TDH-AS1 | Brain_Spinal_cord_cervical_c-1 | susie | 0.19 | 5.50E-07 | -3.74 | 1.83E-04 | 0.0360 |  |
| TDH-AS1 | Brain_Cortex | susie | 0.27 | 3.90E-14 | -3.97 | 7.25E-05 | 0.0293 |  |
| TDH-AS1 | Brain_Hippocampus | susie | 0.29 | 1.20E-12 | -3.97 | 7.23E-05 | 0.0200 |  |
| TDH-AS1 | Brain_Hypothalamus | susie | 0.14 | 5.70E-07 | -3.86 | 1.12E-04 | 0.0224 |  |
| TDH-AS1 | Brain_Substantia_nigra | susie | 0.24 | 1.20E-07 | -3.80 | 1.43E-04 | 0.0227 |  |

**Supplemental Table S5. Colocalization analysis results for eQTL and PGC ASD GWAS in brain and blood tissues.**

| **Gene** | **Tissue** | **PP.H0.abf** | | **PP.H1.abf** | **PP.H2.abf** | **PP.H3.abf** | **PP.H4.abf** | **Sum**  **PPH3**  **PPH4** |
| --- | --- | --- | --- | --- | --- | --- | --- | --- |
| TDH-AS1 | Brain Anterior cingulate corte BA24 | | 2.65E-04 | 2.82E-04 | 5.48E-02 | 5.73E-02 | 8.87E-01 | 0.944 |
| CASP8 | Brain Anterior cingulate corte BA24 | | 2.61E-12 | 5.93E-12 | 5.83E-02 | 1.32E-01 | 8.10E-01 | 0.942 |
| ENSG00000285668 | Brain Anterior cingulate corte BA24 | | 5.93E-15 | 2.19E-12 | 1.86E-03 | 6.85E-01 | 3.13E-01 | 0.998 |
| LRRC37A2 | Brain Anterior cingulate corte BA24 | | 7.60E-18 | 2.82E-15 | 1.85E-03 | 6.86E-01 | 3.12E-01 | 0.998 |
| ARL17A | Brain Anterior cingulate corte BA24 | | 4.10E-08 | 1.52E-05 | 1.87E-03 | 6.91E-01 | 3.07E-01 | 0.998 |
| KANSL1-AS1 | Brain Anterior cingulate corte BA24 | | 1.02E-16 | 3.81E-14 | 2.07E-03 | 7.72E-01 | 2.26E-01 | 0.998 |
| PLEKHM1 | Brain Caudate basal ganglia | | 1.11E-01 | 1.83E-03 | 5.08E-02 | 0.00E+00 | 8.36E-01 | 0.836 |
| LRRC37A2 | Brain Caudate basal ganglia | | 1.42E-25 | 5.24E-23 | 1.85E-03 | 6.85E-01 | 3.14E-01 | 0.999 |
| ARL17A | Brain Caudate basal ganglia | | 1.06E-08 | 3.89E-06 | 1.86E-03 | 6.86E-01 | 3.12E-01 | 0.998 |
| ENSG00000285668 | Brain Caudate basal ganglia | | 9.45E-20 | 3.48E-17 | 1.91E-03 | 7.02E-01 | 2.97E-01 | 0.999 |
| KANSL1-AS1 | Brain Caudate basal ganglia | | 1.87E-24 | 6.96E-22 | 1.97E-03 | 7.32E-01 | 2.66E-01 | 0.998 |
| CASP8 | Brain Cerebellar Hemisphere | | 6.64E-04 | 1.31E-03 | 3.64E-02 | 7.12E-02 | 8.90E-01 | 0.961 |
| RPS23 | Brain Cerebellar Hemisphere | | 4.81E-07 | 1.79E-07 | 1.17E-01 | 4.29E-02 | 8.40E-01 | 0.883 |
| ARL17A | Brain Cerebellar Hemisphere | | 8.67E-07 | 2.98E-04 | 1.97E-03 | 6.75E-01 | 3.23E-01 | 0.998 |
| LRRC37A2 | Brain Cerebellar Hemisphere | | 3.68E-17 | 1.32E-14 | 1.91E-03 | 6.84E-01 | 3.14E-01 | 0.998 |
| ENSG00000285668 | Brain Cerebellar Hemisphere | | 1.25E-10 | 4.46E-08 | 1.92E-03 | 6.85E-01 | 3.13E-01 | 0.998 |
| KANSL1-AS1 | Brain Cerebellar Hemisphere | | 1.07E-15 | 3.87E-13 | 2.33E-03 | 8.40E-01 | 1.58E-01 | 0.998 |
| GABBR1 | Brain Cerebellum | | 1.39E-05 | 5.23E-04 | 1.29E-03 | 4.76E-02 | 9.51E-01 | 0.999 |
| CASP8 | Brain Cerebellum | | 3.15E-14 | 3.28E-14 | 5.94E-02 | 6.08E-02 | 8.80E-01 | 0.941 |
| ATG10 | Brain Cerebellum | | 5.76E-18 | 2.15E-18 | 8.90E-02 | 3.23E-02 | 8.79E-01 | 0.911 |
| TDH-AS1 | Brain Cerebellum | | 7.01E-11 | 1.50E-10 | 4.77E-02 | 1.02E-01 | 8.51E-01 | 0.953 |
| ENSG00000255310 | Brain Cerebellum | | 1.09E-06 | 6.42E-06 | 5.03E-02 | 2.95E-01 | 6.55E-01 | 0.950 |
| ENSG00000236234 | Brain Cerebellum | | 1.26E-06 | 4.42E-04 | 1.89E-03 | 6.64E-01 | 3.33E-01 | 0.997 |
| LRRC37A | Brain Cerebellum | | 7.97E-28 | 3.00E-25 | 1.79E-03 | 6.72E-01 | 3.27E-01 | 0.999 |
| FMNL1 | Brain Cerebellum | | 7.21E-13 | 2.58E-10 | 1.89E-03 | 6.77E-01 | 3.21E-01 | 0.998 |
| PLEKHM1 | Brain Cerebellum | | 5.58E-28 | 2.08E-25 | 1.84E-03 | 6.85E-01 | 3.13E-01 | 0.998 |
| LRRC37A2 | Brain Cerebellum | | 1.92E-34 | 7.18E-32 | 1.83E-03 | 6.86E-01 | 3.12E-01 | 0.998 |
| FAM215B | Brain Cerebellum | | 4.83E-13 | 1.81E-10 | 1.83E-03 | 6.86E-01 | 3.12E-01 | 0.998 |
| ARL17A | Brain Cerebellum | | 9.11E-19 | 3.41E-16 | 1.83E-03 | 6.86E-01 | 3.12E-01 | 0.998 |
| SPPL2C | Brain Cerebellum | | 1.61E-12 | 6.05E-10 | 1.83E-03 | 6.88E-01 | 3.10E-01 | 0.998 |
| KANSL1-AS1 | Brain Cerebellum | | 4.06E-28 | 1.53E-25 | 1.83E-03 | 6.89E-01 | 3.09E-01 | 0.998 |
| ENSG00000285668 | Brain Cerebellum | | 3.19E-27 | 1.19E-24 | 1.86E-03 | 6.94E-01 | 3.04E-01 | 0.998 |
| MAPT-AS1 | Brain Cortex | | 9.32E-04 | 5.36E-04 | 1.12E-02 | 5.48E-03 | 9.82E-01 | 0.987 |
| TDH-AS1 | Brain Cortex | | 1.61E-06 | 3.15E-06 | 5.56E-02 | 1.08E-01 | 8.36E-01 | 0.944 |
| CASP8 | Brain Cortex | | 2.01E-23 | 4.81E-23 | 5.47E-02 | 1.30E-01 | 8.15E-01 | 0.945 |
| LRRC37A2 | Brain Cortex | | 2.61E-27 | 9.64E-25 | 1.86E-03 | 6.87E-01 | 3.11E-01 | 0.998 |
| ARL17A | Brain Cortex | | 3.30E-15 | 1.22E-12 | 1.88E-03 | 6.93E-01 | 3.05E-01 | 0.998 |
| ENSG00000285668 | Brain Cortex | | 3.18E-21 | 1.17E-18 | 1.89E-03 | 6.93E-01 | 3.05E-01 | 0.998 |
| KANSL1-AS1 | Brain Cortex | | 2.02E-31 | 7.50E-29 | 1.87E-03 | 6.93E-01 | 3.05E-01 | 0.998 |
| LRRC37A2 | Brain Frontal Cortex BA10 | | 8.80E-21 | 3.28E-18 | 1.90E-03 | 7.06E-01 | 2.92E-01 | 0.998 |
| ENSG00000285668 | Brain Frontal Cortex BA11 | | 3.60E-15 | 1.33E-12 | 1.85E-03 | 6.86E-01 | 3.12E-01 | 0.998 |
| KANSL1-AS1 | Brain Frontal Cortex BA12 | | 1.81E-22 | 6.75E-20 | 2.43E-03 | 9.09E-01 | 8.84E-02 | 0.997 |
| CASP8 | Brain Frontal Cortex BA13 | | 1.67E-15 | 4.01E-15 | 5.45E-02 | 1.30E-01 | 8.16E-01 | 0.946 |
| TDH-AS1 | Brain Frontal Cortex BA14 | | 9.25E-06 | 1.79E-05 | 4.95E-02 | 9.49E-02 | 8.56E-01 | 0.951 |
| ENSG00000285675 | Brain Frontal Cortex BA15 | | 2.78E-02 | 1.91E-03 | 8.62E-02 | 5.05E-03 | 8.79E-01 | 0.884 |
| ATG10 | Brain Frontal Cortex BA16 | | 1.09E-08 | 4.04E-09 | 1.22E-01 | 4.44E-02 | 8.34E-01 | 0.878 |
| ARL17A | Brain Frontal Cortex BA9 | | 5.93E-13 | 2.21E-10 | 1.85E-03 | 6.87E-01 | 3.11E-01 | 0.998 |
| CASP8 | Brain Hippocampus | | 2.78E-03 | 8.54E-04 | 6.90E-02 | 2.03E-02 | 9.07E-01 | 0.927 |
| TDH-AS1 | Brain Hippocampus | | 2.04E-05 | 8.48E-05 | 5.29E-02 | 2.18E-01 | 7.29E-01 | 0.947 |
| ARL17A | Brain Hippocampus | | 1.22E-06 | 4.29E-04 | 1.93E-03 | 6.78E-01 | 3.20E-01 | 0.998 |
| LRRC37A2 | Brain Hippocampus | | 2.43E-20 | 8.95E-18 | 1.86E-03 | 6.85E-01 | 3.13E-01 | 0.998 |
| ENSG00000285668 | Brain Hippocampus | | 1.02E-14 | 3.74E-12 | 1.87E-03 | 6.88E-01 | 3.11E-01 | 0.999 |
| KANSL1-AS1 | Brain Hippocampus | | 2.34E-20 | 8.66E-18 | 2.40E-03 | 8.89E-01 | 1.08E-01 | 0.997 |
| FAM215B | Brain Hypothalamus | | 1.06E-06 | 3.67E-04 | 1.95E-03 | 6.75E-01 | 3.22E-01 | 0.997 |
| LRRC37A2 | Brain Hypothalamus | | 4.96E-26 | 1.81E-23 | 1.86E-03 | 6.79E-01 | 3.19E-01 | 0.998 |
| ENSG00000285668 | Brain Hypothalamus | | 8.50E-13 | 3.09E-10 | 1.90E-03 | 6.90E-01 | 3.09E-01 | 0.999 |
| ARL17A | Brain Hypothalamus | | 5.11E-11 | 1.87E-08 | 1.89E-03 | 6.91E-01 | 3.07E-01 | 0.998 |
| KANSL1-AS1 | Brain Hypothalamus | | 4.52E-21 | 1.66E-18 | 2.46E-03 | 9.04E-01 | 9.34E-02 | 0.997 |
| TDH-AS1 | Brain Nucleus accumbens basal ganglia | | 6.42E-02 | 1.25E-03 | 4.56E-02 | 0.00E+00 | 8.89E-01 | 0.889 |
| FAM215B | Brain Nucleus accumbens basal ganglia | | 1.38E-04 | 1.39E-04 | 6.09E-02 | 6.06E-02 | 8.78E-01 | 0.939 |
| ARHGAP27 | Brain Nucleus accumbens basal ganglia | | 1.40E-06 | 4.47E-04 | 2.06E-03 | 6.56E-01 | 3.41E-01 | 0.997 |
| LRRC37A2 | Brain Nucleus accumbens basal ganglia | | 1.09E-30 | 3.99E-28 | 1.86E-03 | 6.84E-01 | 3.14E-01 | 0.998 |
| ENSG00000285668 | Brain Nucleus accumbens basal ganglia | | 8.55E-18 | 3.12E-15 | 1.88E-03 | 6.86E-01 | 3.12E-01 | 0.998 |
| ARL17A | Brain Nucleus accumbens basal ganglia | | 6.39E-12 | 2.35E-09 | 1.87E-03 | 6.87E-01 | 3.11E-01 | 0.998 |
| PLEKHM1 | Brain Nucleus accumbens basal ganglia | | 1.73E-07 | 6.28E-05 | 1.91E-03 | 6.93E-01 | 3.05E-01 | 0.998 |
| KANSL1-AS1 | Brain Nucleus accumbens basal ganglia | | 5.97E-28 | 2.20E-25 | 2.03E-03 | 7.50E-01 | 2.48E-01 | 0.998 |
| CTSB | Brain Putamen basal ganglia | | 5.43E-03 | 7.51E-04 | 9.22E-02 | 1.18E-02 | 8.90E-01 | 0.902 |
| LRRC37A2 | Brain Putamen basal ganglia | | 4.99E-23 | 1.82E-20 | 1.88E-03 | 6.85E-01 | 3.13E-01 | 0.998 |
| ENSG00000285668 | Brain Putamen basal ganglia | | 5.48E-19 | 1.99E-16 | 1.88E-03 | 6.85E-01 | 3.13E-01 | 0.998 |
| ARL17A | Brain Putamen basal ganglia | | 1.90E-07 | 6.84E-05 | 1.90E-03 | 6.87E-01 | 3.11E-01 | 0.998 |
| KANSL1-AS1 | Brain Putamen basal ganglia | | 1.75E-29 | 6.41E-27 | 1.91E-03 | 7.00E-01 | 2.98E-01 | 0.998 |
| MAPT-AS1 | Brain Spinal cord cervical c-1 | | 3.20E-02 | 7.44E-03 | 7.94E-03 | 8.94E-04 | 9.52E-01 | 0.953 |
| ENSG00000265547 | Brain Spinal cord cervical c-1 | | 4.49E-06 | 1.58E-03 | 1.89E-03 | 6.65E-01 | 3.31E-01 | 0.996 |
| LRRC37A2 | Brain Spinal cord cervical c-1 | | 8.99E-12 | 3.42E-09 | 1.80E-03 | 6.84E-01 | 3.14E-01 | 0.998 |
| ENSG00000285668 | Brain Spinal cord cervical c-1 | | 5.52E-13 | 2.10E-10 | 1.81E-03 | 6.85E-01 | 3.13E-01 | 0.998 |
| LRRC37A | Brain Spinal cord cervical c-1 | | 2.83E-10 | 1.08E-07 | 1.80E-03 | 6.86E-01 | 3.12E-01 | 0.998 |
| KANSL1-AS1 | Brain Spinal cord cervical c-1 | | 2.10E-13 | 8.05E-11 | 1.80E-03 | 6.87E-01 | 3.11E-01 | 0.998 |
| TDH-AS1 | Brain Substantia nigra | | 1.84E-03 | 1.31E-03 | 5.48E-02 | 3.79E-02 | 9.04E-01 | 0.942 |
| LRRC37A2 | Brain Substantia nigra | | 3.53E-12 | 1.37E-09 | 1.77E-03 | 6.85E-01 | 3.13E-01 | 0.998 |
| KANSL1-AS1 | Brain Substantia nigra | | 5.90E-11 | 2.30E-08 | 1.76E-03 | 6.87E-01 | 3.11E-01 | 0.998 |
| LRRC37A | Brain Substantia nigra | | 1.20E-09 | 4.67E-07 | 1.77E-03 | 6.87E-01 | 3.11E-01 | 0.998 |
| CASP8 | Brain_Amygdala | | 6.64E-04 | 1.31E-03 | 3.64E-02 | 7.12E-02 | 8.90E-01 | 0.961 |
| RPS23 | Brain_Amygdala | | 4.81E-07 | 1.79E-07 | 1.17E-01 | 4.29E-02 | 8.40E-01 | 0.883 |
| ARL17A | Brain_Amygdala | | 8.67E-07 | 2.98E-04 | 1.97E-03 | 6.75E-01 | 3.23E-01 | 0.998 |
| LRRC37A2 | Brain_Amygdala | | 3.68E-17 | 1.32E-14 | 1.91E-03 | 6.84E-01 | 3.14E-01 | 0.998 |
| ENSG00000285668 | Brain_Amygdala | | 1.25E-10 | 4.46E-08 | 1.92E-03 | 6.85E-01 | 3.13E-01 | 0.998 |
| KANSL1-AS1 | Brain_Amygdala | | 1.07E-15 | 3.87E-13 | 2.33E-03 | 8.40E-01 | 1.58E-01 | 0.998 |
| KIZ | Whole Blood | | 3.76E-21 | 1.91E-18 | 1.05E-03 | 5.33E-01 | 4.66E-01 | 0.999 |
| ENSG00000285668 | Whole Blood | | 2.28E-19 | 4.37E-16 | 2.92E-04 | 5.59E-01 | 4.41E-01 | 1.000 |
| LRRC37A | Whole Blood | | 2.78E-83 | 1.42E-80 | 1.30E-03 | 6.63E-01 | 3.35E-01 | 0.998 |
| KANSL1-AS1 | Whole Blood | | 1.13E-77 | 5.71E-75 | 1.63E-03 | 8.22E-01 | 1.76E-01 | 0.998 |

**Supplemental Table S6.** Significant Summary-data-based Mendelian Randomization (SMR) Associations for Autism.

| Gene | topSNP | beta_SMR | se_SMR | pvalue_SMR | pvalue_HEIDI | nsnp_HEIDI |
| --- | --- | --- | --- | --- | --- | --- |
| ARHGAP27 | rs111423688 | 0.181 | 0.060 | 2.69E-03 | 0.16 | 14 |
| ARL17A | rs2532424 | 0.082 | 0.021 | 1.11E-04 | 0.48 | 12 |
| ATG10 | rs112355050 | -0.086 | 0.024 | 2.94E-04 | 0.15 | 20 |
| CASP8 | rs10200279 | 0.069 | 0.019 | 1.95E-04 | 0.28 | 20 |
| CTSB | rs1122182 | 0.123 | 0.041 | 2.93E-03 | 0.64 | 20 |
| FAM215B | rs199535 | 0.086 | 0.023 | 2.41E-04 | 0.86 | 10 |
| FMNL1 | rs62062283 | -0.113 | 0.028 | 6.35E-05 | NA | NA |
| GABBR1 | rs3025626 | 0.195 | 0.048 | 4.10E-05 | 0.55 | 8 |
| KANSL1-AS1 | rs199535 | 0.067 | 0.018 | 1.19E-04 | 0.32 | 11 |
| LRRC37A2 | rs2942166 | 0.064 | 0.015 | 3.60E-05 | 0.72 | 8 |
| MAPT-AS1 | rs369332489 | -0.114 | 0.033 | 4.87E-04 | 0.54 | 15 |
| PLEKHM1 | rs62065444 | 0.173 | 0.052 | 9.67E-04 | 0.10 | 16 |
| SPPL2C | rs2243967 | 0.112 | 0.028 | 4.98E-05 | 0.70 | 8 |

**Supplemental Table S7.** Phenotypes associated with candidate targets of ASD in knockout mice model.

| ***Gene*** | ***Model*** | ***Genetic Category*** | ***Genotype ID*** | ***Specific Nervous System Phenotype*** | ***Other Abnormal Phenotypes*** | ***Reference***  ***(PMID)*** | |  |
| --- | --- | --- | --- | --- | --- | --- | --- | --- |
| ARHGAP27 |  | Targeted (Conditional ready, Null/knockout, Reporter) (Cell Line) | MGI:4419662 | |  |  | |  |
| ARL17A | No characterized mouse model available |  |  |  |  |  | |  |
| ATG10 |  | Targeted (Null/knockout, Reporter) | MGI:4452822 | |  |  | |  |
| CASP8 | Homozygous knock-out, non-conditional | Targeted (Null/knockout) | MGI:5546196, MGI:3609638 | wavy neural tube, abnormal neural tube morphology, kinked neural tube | cardiovascular, embryo, mortality/aging, cellular, hematopoietic, homeostasis, muscle, | 12654726,  12404118 | |  |
| CTSB | Homo and Heterozygous knock-out, non-conditional | Targeted (Null/knockout) | MGI:2182129 | | cellular, digestive/alimentary, endocrine/exocrine, homeostasis, immune | 9539769 | |  |
| FAM215B | No characterized mouse model available |  |  |  |  |  | |  |
| FMNL1 | conditional knock-out | Targeted (Null/knockout) | MGI:6119535 | | cardiovascular, cellular, hematopoietic, immune, liver/biliary | [28348104](https://www.ncbi.nlm.nih.gov/entrez/query.fcgi?cmd=Retrieve&db=PubMed&list_uids=28348104&dopt=Abstract) | |  |
| GABBR1 | Homo and Heterozygous knock-out, non-conditional | Targeted (Null/knockout) | MGI:2159352, MGI:2159354, MGI:3512742, MGI:3665432, MGI:3665433 | audiogenic seizures, tonic-clonic seizures, sporadic seizures, abnormal axon morphology, abnormal somatic nervous system morphology, abnormal myelination, abnormal nervous system electrophysiology, abnormal synaptic transmission, abnormal inhibitory postsynaptic potential, increased prepulse inhibition, abnormal neuron physiology, abnormal CNS synaptic transmission, abnormal excitatory postsynaptic currents, reduced long-term potentiation | behavior, homeostasis, nervous system, reproductive, growth/size/body, mortality/aging | 11498050,  11414794,  15493018,  16701209,  16701209 |  |  |
| KANSL1-AS1 | No characterized mouse model available |  |  |  |  |  | |  |
| LRRC37A |  | Endonuclease-mediated (Null/knockout) | MGI:7519113 | |  |  | |  |
| LRRC37A2 | No characterized mouse model available |  |  |  |  |  | |  |
| MAPT-AS1 | No characterized mouse model available |  |  |  |  |  | |  |
| PLEKHM1 | Homozygous knock-out, non-conditional | Targeted (Null/knockout, Reporter) | MGI:5513810 | abnormal optic disk morphology, decreased prepulse inhibition | adipose, growth/size/body, homeostasis, limbs/digits/tail, renal/urinary, skeleton, vision/eye |  | |  |
| SPPL2C | Homozygous knock-out, non-conditional | Targeted (Null/knockout) | MGI:6358787 | | cellular, endocrine/exocrine, reproductive | [30733280](https://www.ncbi.nlm.nih.gov/entrez/query.fcgi?cmd=Retrieve&db=PubMed&list_uids=30733280&dopt=Abstract) | |  |
| TDH-AS1 | No characterized mouse model available |  |  |  |  |  | |  |
|  |  |  |  |  |  |  | |  |

**Supplementary Figure S1.** KEGG pathway enrichment analysis of ASD-risk targets with 4 enriched pathways. Purple represents ATG10, yellow represents CASP8, red represents CTSB and blue represents PLEKHM1.


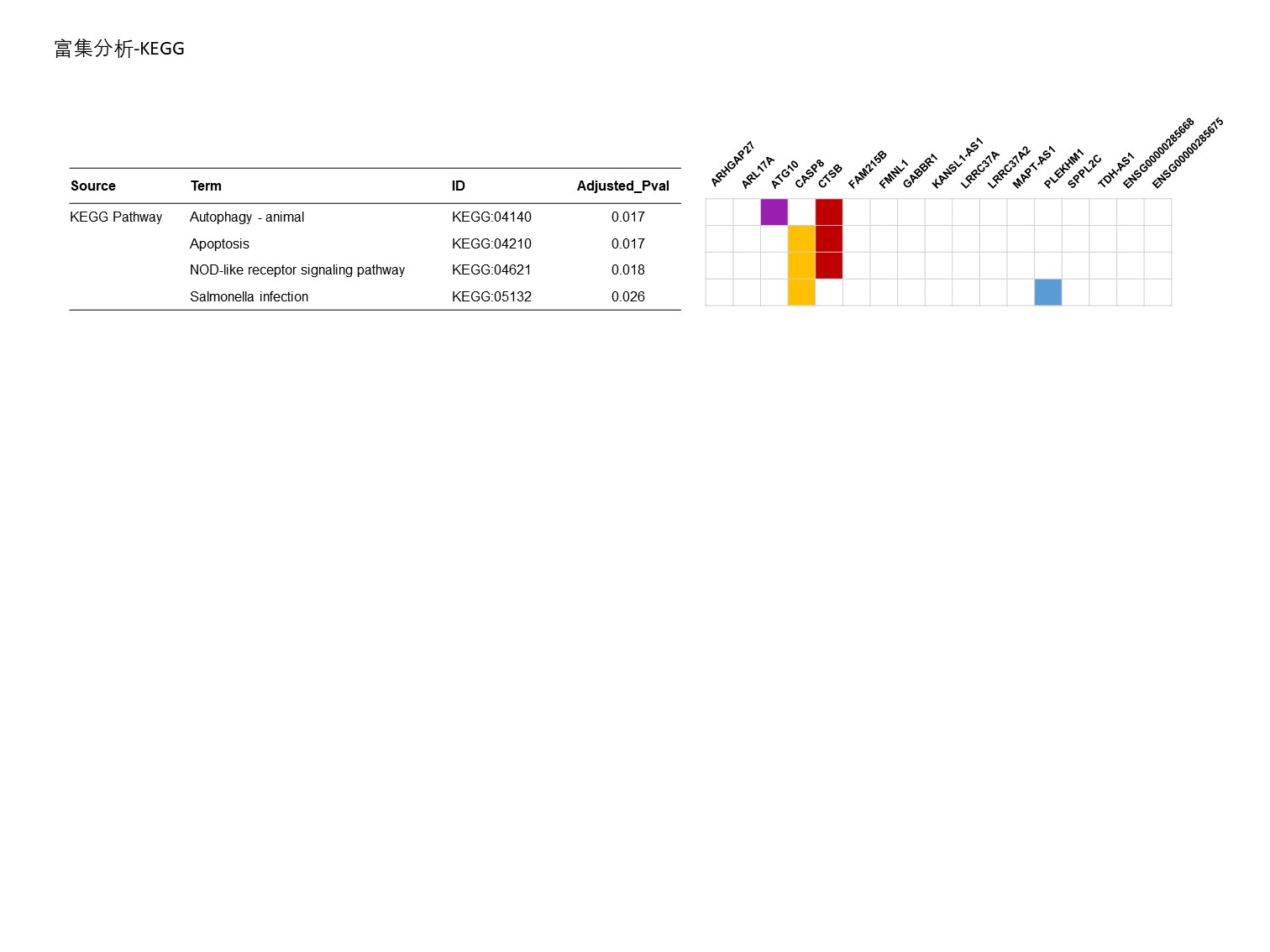


**Supplementary Figure S2.** GO Cellular Component enrichment analysis of ASD-risk targets with 22 enriched terms. Purple represents ATG10, yellow represents CASP8, red represents CTSB, cyan represents FAM215B, green represents GABBR1, blue represents PLEKHM1 and orange represents SPPL2C.


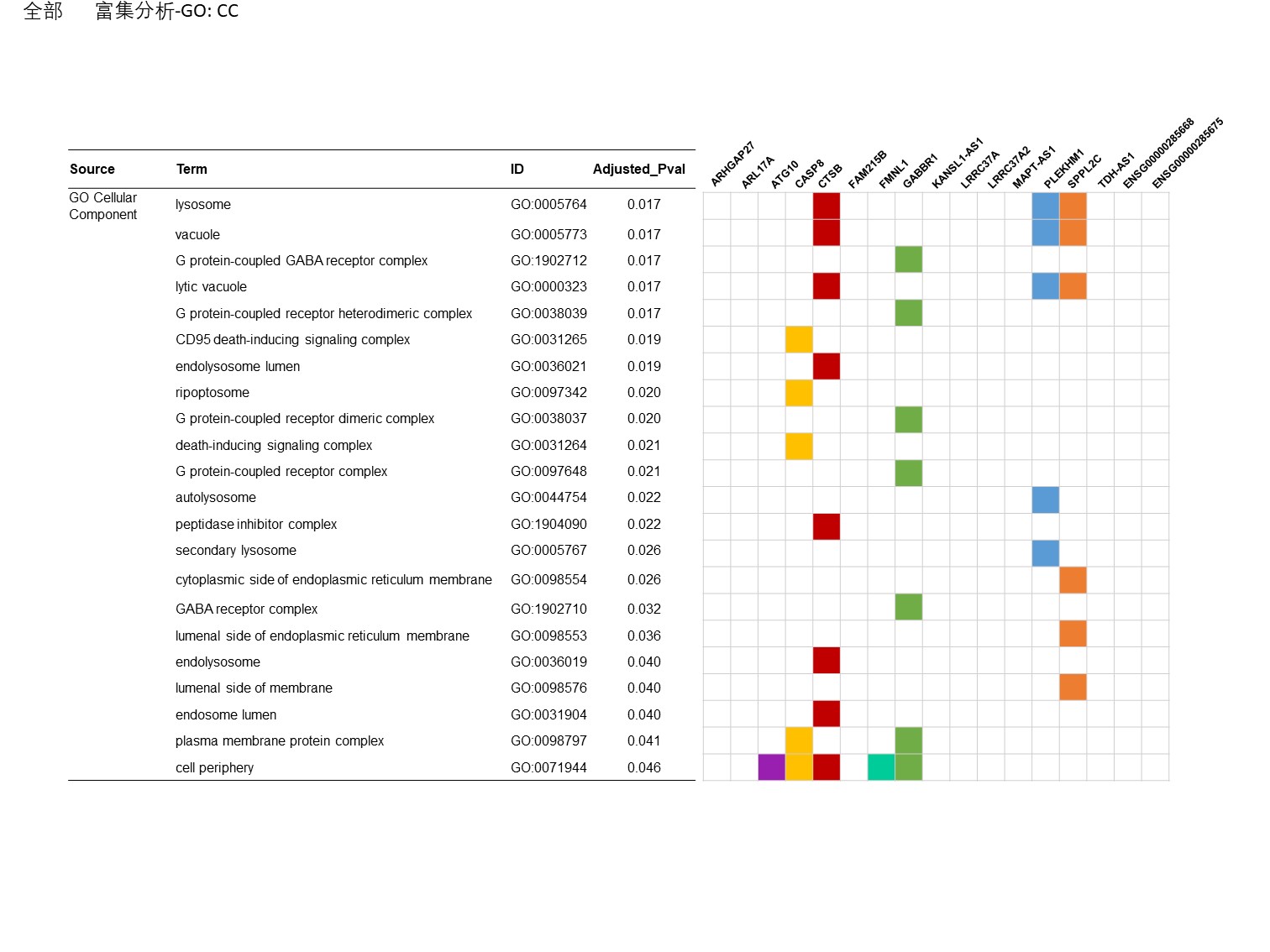


**Supplementary Figure S3**. GO Molecular Function enrichment analysis of ASD-risk targets with 22 enriched terms. Dark blue represents ARHGAP27, purple represents ATG10, yellow represents CASP8, red represents CTSB, cyan represents FAM215B, green represents GABBR1and orange represents SPPL2C.


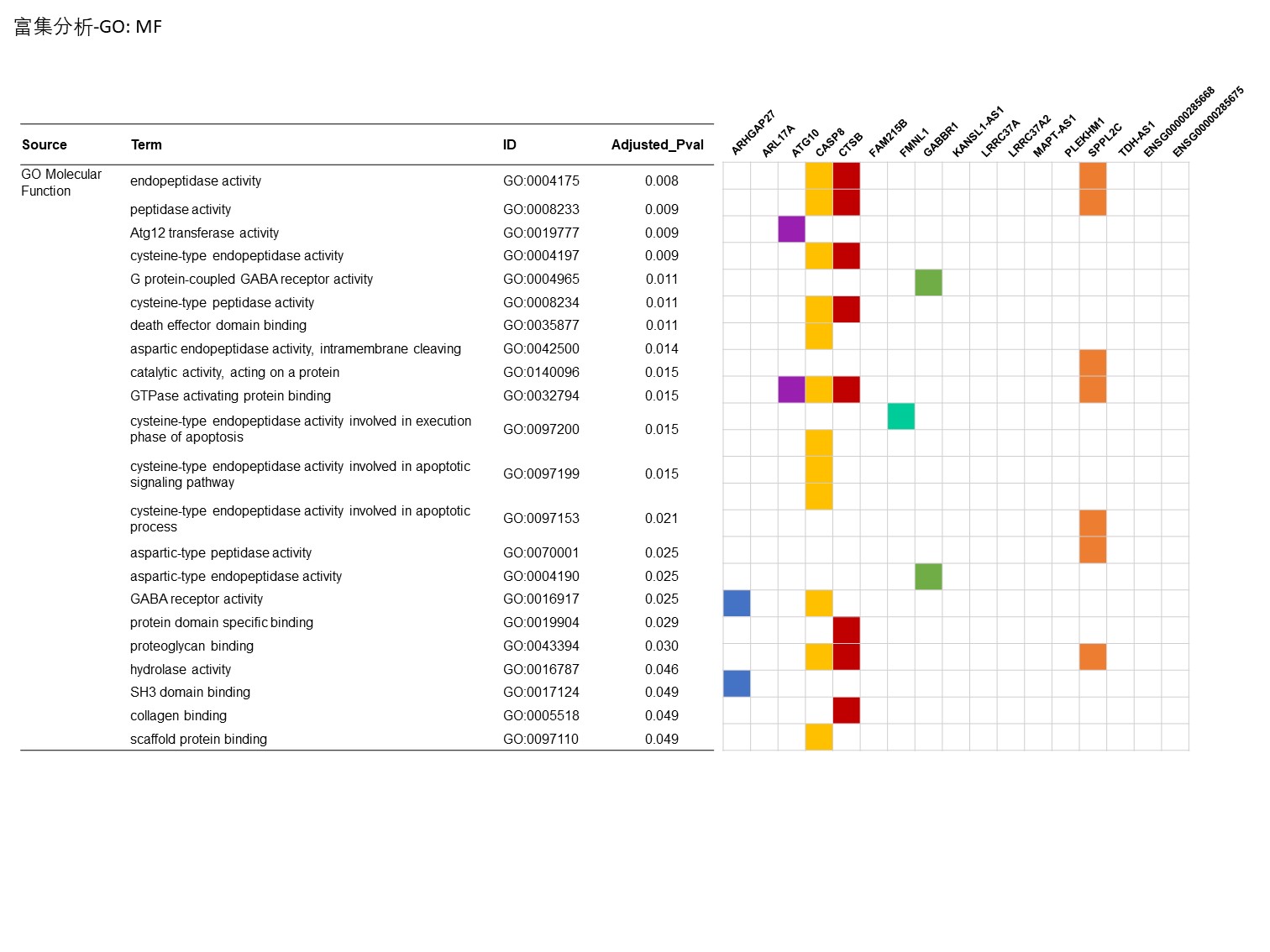

Supplement: Supplemental_Materials-revised_bbae353 [file supplemental_materials-revised_bbae353.docx]
